# Excess mortality during the COVID-19 pandemic in low-and lower-middle-income countries: a systematic review and meta-analysis

**DOI:** 10.1186/s12889-024-19154-w

**Published:** 2024-06-20

**Authors:** Jonathan Mawutor Gmanyami, Wilm Quentin, Oscar Lambert, Andrzej Jarynowski, Vitaly Belik, John Humphrey Amuasi

**Affiliations:** 1 School of Public Health, Kwame Nkrumah University of Science and Technology, Kumasi, Ghana; 2 German West-African Centre for Global Health and Pandemic Prevention, Berlin, Germany; 3 Global Health and Infectious Diseases Research Group, Kumasi Centre for Collaborative Research in Tropical Medicine, Kumasi, Ghana; 4 Department of Health Care Management, Technische Universität Berlin, Berlin, Germany; 5 Chair of Planetary & Public Health, University of Bayreuth, Bayreuth, Germany; 6 Department of Veterinary Medicine, Freie Universität Berlin, Berlin, Germany; 7 Bernhard Nocht Institute for Tropical Medicine, Hamburg, Germany

**Keywords:** Excess mortality, COVID, 19 pandemic, Low- and lower-middle-income countries

## Abstract

**Background:**

Although the COVID-19 pandemic claimed a great deal of lives, it is still unclear how it affected mortality in low- and lower-middle-income countries (LLMICs). This review summarized the available literature on excess mortality during the COVID-19 pandemic in LLMICs, including methods, sources of data, and potential contributing factors that might have influenced excess mortality.

**Methods:**

We conducted a systematic review and meta-analysis on excess mortality during the COVID-19 pandemic in LLMICs in line with the Preferred Reporting Items for Systematic Review and Meta-Analysis (PRISMA) 2020 guidelines We searched PubMed, Embase, Web of Science, Cochrane Library, Google Scholar, and Scopus. We included studies published from 2019 onwards with a non-COVID-19 period of at least one year as a comparator. The meta-analysis included studies reporting data on population size, as well as observed and expected deaths. We used the Mantel–Haenszel method to estimate the pooled risk ratio with 95% confidence intervals. The protocol was registered in PROSPERO (ID: CRD42022378267).

**Results:**

The review covered 29 countries, with 10 countries included in the meta-analysis. The pooled meta-analysis included 1,405,128,717 individuals, for which 2,152,474 deaths were expected, and 3,555,880 deaths were reported. Calculated excess mortality was 100.3 deaths per 100,000 population per year, with an excess risk of death of 1.65 (95% CI: 1.649, 1.655, *p* < 0.001). The data sources used in the studies included civil registration systems, surveys, public cemeteries, funeral counts, obituary notifications, burial site imaging, and demographic surveillance systems. The primary techniques used to estimate excess mortality were statistical forecast modelling and geospatial analysis. One out of the 24 studies found higher excess mortality in urban settings.

**Conclusion:**

Our findings demonstrate that excess mortality in LLMICs during the pandemic was substantial. However, estimates of excess mortality are uncertain due to relatively poor data. Understanding the drivers of excess mortality, will require more research using various techniques and data sources.

**Supplementary Information:**

The online version contains supplementary material available at 10.1186/s12889-024-19154-w.

## Introduction

Only six viruses within the coronavirus family, namely 229E, NL63, OC43, HKU1, SARS-CoV, and MERS-CoV, have been known to cause respiratory tract infections in humans [1]. The SARS-CoV-2 virus, identified in 2019 as the cause of COVID-19, emerged in Wuhan, China [2]. Despite containment efforts, the virus spread globally, leading the World Health Organization (WHO) to declare it a pandemic in March 2020 [3]. To date, over 6.5 million deaths and 623 million infections have been reported worldwide, with Africa recording nearly 9 million cases and over 173,000 deaths [4].

Numerous non-pharmaceutical interventions were adopted globally to combat COVID-19, such as lockdowns and mask mandates [5–7]. While these measures aimed to reduce the transmission of the virus, [8, 9] may have inadvertently increased mortality among chronically ill patients by hindering timely medical care access [10, 11]. Additionally, the pandemic response contributed to higher fatalities from domestic violence, suicide, and mental health issues [9, 12, 13].

Confirmed COVID-19 deaths alone may not fully reflect the pandemic's impact [14]. Excess mortality offers a more comprehensive view, capturing both direct and indirect effects. As per the World Health Organization (WHO), excess mortality is the difference between actual deaths during a crisis and expected deaths without it [15], encompassing COVID-19-related deaths and those indirectly influenced by the pandemic, including socio-economic challenges like compromised food security, disruptions in supply chains, and limited access to healthcare [16–18].

Studies have shown that the pandemic exacerbated food insecurity due to lockdowns and economic downturns, which affected the nutritional status and health outcomes of vulnerable populations. Additionally, disruptions in healthcare services led to delays in treatment for chronic conditions and reduced access to essential medical care, further increasing mortality. Mental health issues and increased domestic violence during lockdowns also contributed to higher death rates indirectly associated with the pandemic. These multifaceted impacts highlight the necessity of assessing excess mortality to gain a full understanding of the pandemic's toll, particularly in low- and lower-middle-income countries (LLMICs), where healthcare systems and social safety nets are often less robust. The estimated excess mortality rate from COVID-19 could be 5 to 25-fold higher than reported COVID-19 mortality rates [14].

Understanding and accurately reporting mortality statistics is crucial for global health policy and resource allocation. In low- and lower-middle-income countries (LLMICs), mortality reporting remains a significant challenge. These countries often face systemic challenges, including incomplete civil registration systems, and under-resourced statistical offices, which contribute to incomplete or inaccurate mortality data. Hence knowledge on excess mortality during the COVID-19 pandemic in LLMICs remains limited [19–22]. Vital registration systems and other data sources are often incomplete or inaccurate, lacking routine mortality reporting [5, 20, 23]. To address these limitations, various methods like data interpolation and extrapolation have been proposed [24]. Innovative approaches such as using satellite imagery to track new graves and participatory epidemiology have also been employed to estimate excess mortality [25–27] and these unique circumstances and innovative solutions emerging from LLMICs require focused attention.

In addition, to estimating excess mortality using available data, Shang et al. observed a higher pooled excess mortality in developing countries compared to developed ones but did not delve into specific LLMIC results or assess methodologies and data in these contexts [28]. This systematic review and meta-analysis presents a focused and current summary of excess mortality literature in LLMICs. This study helps to fill a critical gap in the literature by systematically reviewing and analyzing excess mortality in LLMICs during the COVID-19 pandemic. This will not only enhance our understanding of the pandemic's true impact but also support the development of more effective public health responses in these vulnerable regions.The objectives included summarizing existing studies on excess mortality during the COVID-19 pandemic, describing estimation methods and data sources, and identifying drivers of excess mortality in these settings.

## Methods

### Settings

This systematic review and meta-analysis focused on studies from low- and lower-middle-income countries.

This review, guided by the Preferred Reporting Items for Systematic Reviews and Meta-Analyses (PRISMA) [29], focused on estimating excess mortality levels, examining the methodologies and data used for estimation, and identifying factors influencing excess mortality in LLMICs. Quantitative methods were utilized to conduct a meta-analysis, providing a summary estimate of the excess mortality.

### Protocol registration

The protocol for conducting this systematic review and meta-analysis was registered in the International Prospective Register of Systematic Reviews (PROSPERO) (ID: CRD42022378267).

### Searches

We conducted searches in electronic bibliographic databases including PubMed, Embase, Web of Science, Cochrane Library, Google Scholar, and Scopus. Additionally, we reviewed the reference lists of included studies and relevant publications. The search strategy comprised terms related to key review concepts: COVID-19 and/or SARS-CoV-2, excess mortality, and low- and lower-middle-income countries. Each term was operationalized with various synonyms and tailored for specific databases. The search strategy used Medical Subject Headings (MeSH) terms and involved key terms with the appropriate Boolean operators (AND, OR) to ensure comprehensive coverage.

No language restrictions were applied, and the searches were restricted to studies published between 2019 and the date of the searches. In September 2023, the searches were rerun before the final analyses, resulting in additional studies for inclusion.

### Study selection procedures

The inclusion and exclusion criteria were defined based on the Participants, Intervention/Exposure, Comparator, and Outcome (PICO) framework, as detailed below:

#### Participants/population

The review included population-level or cohort studies from LLMICs, independent of the administrative level (district, region, nation). Facility-based studies were considered to examine covariates and the methods used, but disease-specific studies were excluded.

#### Intervention(s)/exposure

The exposure of interest was the COVID-19 pandemic. This referred to the period from when the WHO declared COVID-19 a pandemic on March 11, 2020, to the most current wave of COVID-19 infection that was reported in the population under review.

#### Comparator(s)/control

The comparator in the estimation of the excess mortality was all-cause mortality in the non-COVID-19 period (registered or estimated). This comparator period included data from at least one year before March 2020.

#### Main outcome

The main review outcome was excess mortality in the population under investigation.

#### Additional outcome

Additional outcomes included the methods and data sources used in estimating excess mortality and factors that influenced excess mortality in LLMICs.

### Eligibility criteria

1. Articles that reported on excess mortality with the COVID-19 pandemic as the exposure of interest
2. Articles conducted in Low and Lower-Middle Income Countries as defined by the World Bank
3. Studies published between the years 2019 and to date
4. Population-level, cohort studies or facility-based studies, independent of the administrative level (district, region, nation)
5. Studies with a comparator of all-cause mortality in the non-COVID-19 period being at least one year before March 2020

### Study inclusion

Two independent investigators (JMG and OL) used the eligibility criteria to select studies for inclusion in the review. Any disagreement was resolved by discussion and/or a third reviewer (WQ) was consulted for a consensus to be reached. A meta-analysis was conducted for a subset of the included studies in the review. Studies were included in the meta-analysis only if they provided the following information: a clearly defined estimate for excess mortality, a documented method for estimating excess mortality, a specified population size for the study, as well as an observed, and expected death count for the period reported.

### Data extraction

We extracted the following data: author (s), publication year, study country, study period, World Bank income level, estimated excess mortality, disaggregated results for differences in socio-economic groups, estimated and registered COVID-19 mortality, mortality data sources, methods used to estimate excess mortality, identified drivers of excess mortality, type of population (geographical region, cohort), and population baseline characteristics. Mendeley Desktop Version 1.19.8 was used to identify duplicate records.

### Measures of effect

The review’s primary outcome was estimated excess mortality as reported in primary studies. Studies that did not indicate the expected (i.e. baseline) deaths and the observed/estimated deaths were not included in the meta-analysis. Secondary outcomes included methods for estimating excess mortality, disaggregated measures of excess mortality (e.g. mortality by socio-economic status) and factors influencing excess mortality.

### Data analysis and synthesis

Reported estimates of excess mortality were summarized in tabular format and synthesized narratively. The methods and data that were used for estimating excess mortality and identifying factors that influenced excess mortality, and the socioeconomic disparities in the estimates of excess mortality were summarized and synthesized into thematic narratives.

A meta-analysis was conducted to estimate the rate of excess mortality in LLMICs. Data analysis was conducted using StataSE 16 statistical software from StataCorp, College Station, Texas, USA.. Mortality rates estimated before and during the pandemic were calculated and summarized. The Mantel–Haenszel random-effects method was adopted to estimate the pooled risk ratio at 95% confidence intervals (CIs) and heterogeneity among the studies was estimated using *I*^2^ values. The *I*^2^ quantified the degree of heterogeneity in the meta-analysis.

### Sensitivity analysis

Sensitivity analyses were carried out to investigate how non-eligible research may have an impact on risk differences. This was accomplished by running the data through a meta-analysis twice. For studies that did not have full details based on the eligibility criteria, first, we included all studies and second, only included those that were known to be eligible. Only studies that were known to be eligible were included in the final meta-analysis.

### Risk of bias (quality) assessment

The quality of the included studies was assessed using appropriate tools. Quality assessment was performed by two independent reviewers based on the Newcastle- Ottawa Scale (NOS) score and any disparity was solved by discussion and/or consulting a third reviewer (Appendix 1). In this assessment, all studies included in the review and meta-analysis were at minimal risk of bias. In addition to the NOS score, we also considered the methodological rigor of each study, including factors such as study design, sample size, and data collection methods. This comprehensive assessment ensured a thorough evaluation of the quality of the included studies and provided confidence in the robustness of our findings.

## Results

### Study selection procedures

**Fig. 1:**
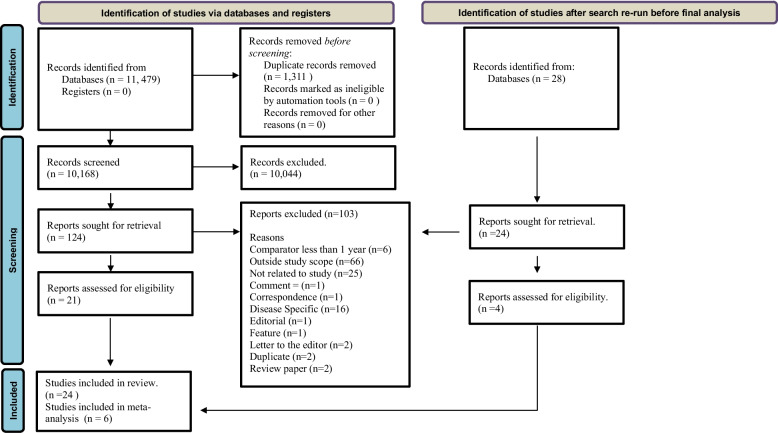
Flow diagram of the study selection procedure

Figure 1 summarizes the results of the study search and selection process. A total of 10,196 studies were identified in the databases after removal of duplicates. During title and abstract screening, 10,068 were excluded, leaving 129 studies for full-text review, of which, 24 studies were included in the systematic review and 6 in the meta-analysis.

The main reasons for exclusion in the review were (1) Reports outside the study scope, (2) Studies not related to review objectives, (3) estimation of excess mortality among patients with a specific disease instead of a population and/or cohort, and (4) the use of a comparator which was less than 1 year in the estimation of the expected number of deaths in the calculation of excess mortality. The main reasons for exclusion from the meta-analysis were that studies did not specify the population size, the number of expected deaths (all-cause mortality), the number of observed deaths, or the methods for estimating excess mortality.

### Characteristics of included eligible studies

**Fig. 2:**
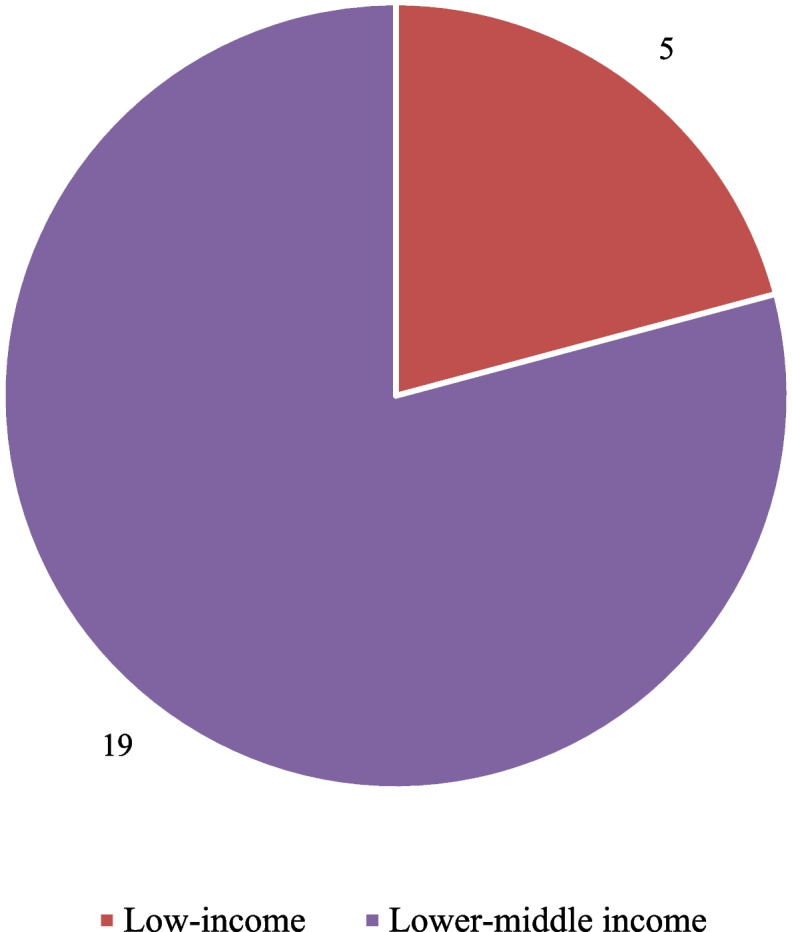
Number of studies classified by World Bank income level

**Fig. 3:**
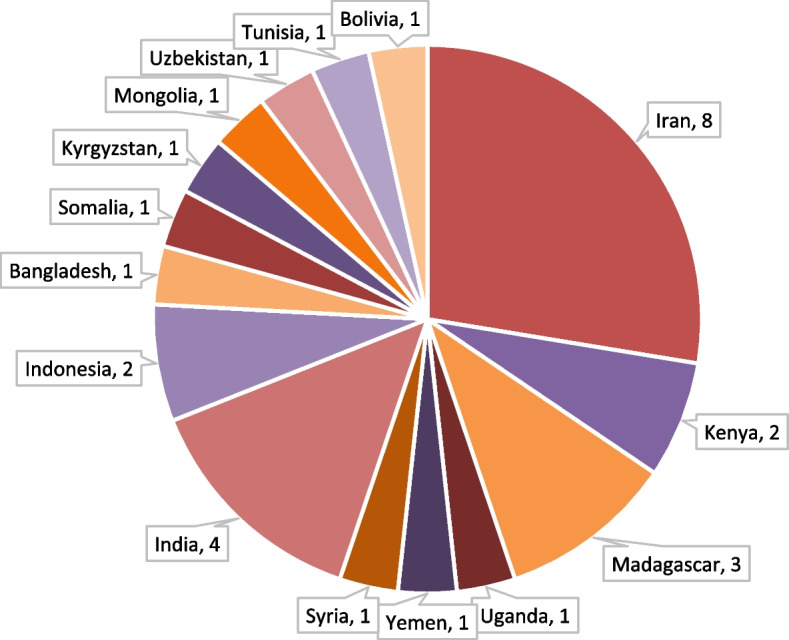
Countries and their represented number of included number of studies

The characteristics of the 24 included studies are summarized in Table 1. Studies were published between 2020 and 2023 but most were published in 2021 (13 studies). Five studies were conducted in low-income countries and 19 in lower-middle-income countries (Fig. 2). Most of the studies were conducted in Asia, including Iran (7). India (4), Bangladesh (2), and Indonesia (2). There were 6 studies from Africa and none from Latin America or the Caribbean. Sanmarchi et al. [30] reported estimates from 5 countries, making it a total of 29 countries in the review (Fig. 3).

**Table 1:** Study characteristics of included studies

Authors (first author)	Year	Country	World Bank Income Level	Type of population	Sex	Age group	Sources of mortality data	Data years included	Method/model used to estimate excess mortality
Hedstrom et al. [31]	2021	Uganda	Low income income	Provider-based	Both	0—92	Electronic database of all neonatal admissions	2018—2020	Proportion of infants who died to the total number of infants admitted to the unit in the same period
Besson et al. [32]	2021	Yemen	Low income income	Governorate	Both	Not reported	Burial ground quntification	2016—2020	Geospatial analysis—techniques to manually identify new grave plots and measure changes in burial surface area over a period
Hanifi et al. [33]	2021	Bangladesh	Lower middle income	District	Both	0—65 +	Health and Demographic Surveillance System	2015—2020	Cox proportional hazard models
Jha et al. [34]	2022	India	Lower middle income	National	Both	Not reported	National survey data + health facility deaths	2018—2021	Not specified
Tadbiri et al. [35]	2020	Iran	Lower middle income	National	Both	Not reported	National Organization for Civil Registration	2013—2020	linear regression model
Ghafari et al., [36]	2022	Iran	Lower middle income	National	Both	0—80 +	National Organization for Civil Registration	2015—2019	Quantitative framework
Otiende et al. [37]	2021	Kenya	Lower middle income	National	Both	0—74	Health and Demographic Surveillance System	2003—2018	Negative bionomial model
Watson et al. [38]	2021	Syria	Low income income	National	Both	Not reported	Daily mortality and incidence data from the Syrian Ministry of Health + Excess all-cause mortality data from a statement by the Damascus governorate + Obituary notification data from Facebook page	2017—2021	Mathematical model of COVID-19 transmission
Safavi-Naini et al. [39]	2022	Iran	Lower middle income	National	Both	0—85 +	National Organization of Civil Registration	2013—2021	Auto-Regressive Integrated Moving Average (ARIMA) or exponential smoothing model
Lewnard et al. [18]	2021	India	Lower middle income	state	Both	0—80 +	Civil Registration System	2016—2021	Generalised linear models with a negative binomial link function
Ghafari et al. [40]	2021	Iran	Lower middle income	Province level	Both	Not specified	National Organization for Civil Registration	2015—2020	Piecewise linear regression model
Rasambainarivo et al. [41]	2021	Madagascar	Low income income	District	Both	0—90	Death registers	2010—2019	Autoregressive quasi Poisson and an autoregressive negative binomial regression model
Wijaya et al. [42]	2022	Indonesia	Lower middle income	District	Both	Not reported	Jakarta's Open Data a monthly basis from March 2020, when COVID-19 was officially declared to have transmitted in Indonesia, to December 2020	2018—2020	linear mixed model
Leffler et al. [43]	2022	India	Lower middle income	Regions	Both	Not reported	Figures published by regional governments and Indian journalists + government hospital data + funeral counts + handwritten death registers	2019—2021	linear regression
Barnwal et al. [23]	2021	Bangladesh	Lower middle income	District	Both	0—80	Census/Survey—Primary data	2019—2021	Multilevel regression model (full bayesian model)
Warsame et al. [22]	2021	Somalia	Low income income	Regions	Both	Not reported	Burial ground quntification	2017—2019	Geospatial analysis—quntify number of burials
Acosta et al. [24]	2021	India	Lower middle income	District	Both	Not reported	Death registers	2019—2020	Using a model fit
Elyazar et al. [44]	2020	Indonesia	Lower middle income	District	Both	Not reported	Burials in public cemeteries + civil death registration + health authority death registration	2015—2020	Counts estimation
Sanmarchi et al. [45]	2021	Kyrgyzstan, Mongolia, Uzbekistan, Tunisia, Bolivia	Low and Lower middle income	National	Both	Not reported	Aggregated country-level data on population and COVID-19 overall confirmed cases, deaths, and testing as of December 31, 2020, from World Data	2015—2020	Negative binomial regression models
Esmaeilzadeh et al. [46]	2023	Iran	Lower middle income	Province	Both	Not reported	Monthly vital statistics reports	2020 – 222	Univariate time series analysis
Oduor et al. [47]	2023	Kenya	Lower middle income	District	Both	1 – 65	Infectious Disease Surveillance	2016 – 2021	Negative binomial regression model
Ebrahimoghli et al. [48]	2023	Iran	Lower middle income	National	Both	0 – 85	National Organization for Civil Registration	2019 -2021	interrupted time-series design
Rabarison et al. [49]	2023	Madagascar	Lower middle income	Region	Both	Not specified	Electronic database of all admissions	2010 – 2021	The proportion who died divided by the total number of patients admitted

For the meta-analysis, 10 countries were included from 6 studies. In 7 countries, the observed deaths were higher than expected ([India (2), Iran (1), Kyrgyzstan (1), Uzbekistan (1), Tunisia(1), and Bolivia (1)]. In three countries (Indonesia, Kenya and Mongolia), negative excess mortality was recorded, thus the observed deaths were lower than the number expected in the absence of the pandemic.

### Estimate of excess mortality in LLMICs

**Fig. 4:**
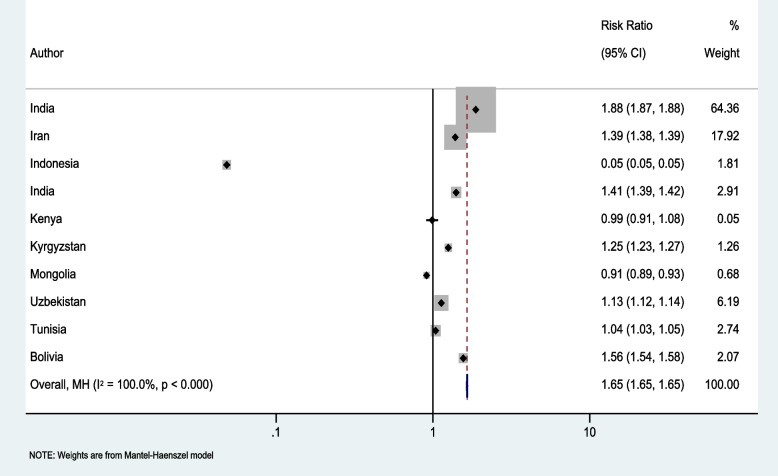
Adjusted Pooled estimate of excess mortality

Table 2 provides an overview of population and mortality data reported by the studies included in the meta-analysis. During the COVID-19 pandemic, of the total 1,398,858,717 individuals/populations, 3,555,880 all-cause deaths were reported, while 2,152,474 deaths were expected from the eleven countries. The pooled excess mortality was 100.3 deaths per 100,000 population. The excess risk of death was 1.65 (95% CI: 1.649, 1.655 *p* < 0.001). There was a high heterogeneity as indicated by the *I*^2^ of 100% among the studies (Fig. 4).

**Table 2:** Studies included in meta-analysis (*n* = 10)

Country	Data years included	Population	Expected deaths	Expected Alive	Observed deaths	Observed Alive	Excess deaths
Iran	2013—2021	83,748,183	385,778	83,362,405	535,570	83,212,613	149,792
Indonesia	2018—2020	10,534,517	38,865	10,495,652	1,881	10,532,636	-36,984
India	2019—2021	4,995,398	62,690	4,932,708	88,107	4,907,291	25,417
Kenya	2003—2018	300,000	1,012	298,988	1,000	299,000	-12
Kyrgyzstan	2015—2020	6,524,013	27,135	6,496,878	33,995	6,490,018	6,860
India	2016—2021	1,232,519,753	1,385,409	1,231,134,344	2,600,000	1,229,919,753	1,214,591
Mongolia	2015—2020	3,278,523	14,554	3,263,969	13,258	3,265,265	-1,296
Uzbekistan	2015—2020	33,467,125	133,298	33,333,827	150,808	33,316,317	17,510
Tunisia	2015—2020	11,818,182	59,078	11,759,104	61,509	11,756,673	2,431
Bolivia	2015—2020	11,673,023	44,655	11,628,368	69,752	11,603,271	25,097

In 7 countries, the observed deaths were higher than expected, whilst, in three countries, negative excess mortality was recorded, thus the observed deaths were lower than the number expected in the absence of the pandemic.

### Methods in estimating excess mortality in LLMICs

The 24 articles used four distinct methods/study designs to determine excess mortality. The largest group of studies (15 articles) used retrospective data of already existing mortality datasets [Bangladesh (1), Iran (5), India (4), Kenya(1); Syria(1), Madagascar(1), Indonesia(1), Uganda(1)] to estimate excess mortality. Two studies used quantification of burial sites by observing the increase in the number of burial grounds to estimate excess mortality [Yemen(1), Somalia(1)]. One study used a cross-sectional survey through a household census (Bangladesh) and another used grey literature (use of already published figures from journalists and organizations) (India) to estimate excess mortality.

Concerning the source of data, four studies used more than one data source to estimate excess mortality. This included burials in public cemeteries + civil death registration + health authority death registration (Indonesia), daily mortality/incidence data from the Syrian Ministry of Health + Excess all-cause mortality data from a statement by the Damascus governorate + obituary notification data from Facebook page (Syria), National survey data + health facility deaths Jha et al. [49] (India) and figures published by regional governments and Indian journalists + government hospital data + funeral counts + handwritten death registers (India).

All other studies relied on only one data source. Five studies used National Civil Registration Data (4 studies from Iran and 1 India). Two studies each used the Health and Demographic Surveillance System (Kenya and Bangladesh), death registers (India and Madagascar) and imaging of burial sites/grounds (a study each from Yemen and Somalia). One study (in Bangladesh) used only primary data (census/survey) data and another study (in Iran and Indonesis) used Bureau of Vital Statistics data to estimate excess mortality.

Studies used several different methods to determine the expected deaths that were used to calculate excess mortality. Twelve studies used modelling techniques to estimate excess mortality. Of these, five studies used linear regression [India(2), Indonesia(1); Iran(2)], two studies used auto-regression modelling techniques. Two other studies (In Madagascar and Iran) used geospatial analysis which involves identifying new grave plots and measuring changes in burial surface area over a period ( In Yemen and Somalia) and two studies used estimation of death counts (In Uganda and Indonesia). Other modelling techniques used included Cox proportional hazard models, Auto-Regressive Integrated Moving Average, model fit, multilevel regression model (full bayesian model).

### Factors influencing excess mortality in LLMICs

In assessing the factors that might have influenced excess mortality, of the 24 studies, only one (in India) reported differences in mortality between rural and urban areas. They found that excess deaths in the first wave of the pandemic were concentrated in urban areas, while deaths in the second wave affected both urban and rural areas. Other studies speculated what could have caused excess morality without empirical evidence in their data. No study reported disaggregated information by socio-economic status.

### Discussion of key findings

This is the first systematic review and meta-analysis of studies estimating excess mortality during the COVID-19 pandemic in low- and lower-middle-income countries (LLMICs), exploring methods in estimating excess mortality and the factors that might have influenced excess morality in LLMICs.

The results of the meta-analysis indicate that excess mortality in LLMICs was substantial. There were an estimated 1,403,406 excess deaths in the 10 countries covered by the included studies, representing 100.3 excess deaths per 100,000 population or a 1.65 excess risk of death (95% CI: 1.649, 1.655 *p* < 0.001) during the pandemic. Expected deaths were mostly estimated based on secondary data analysis. Other studies quantified an increase in burial grounds and other household surveys. This review identified only one study that assessed factors associated with excess mortality. According to that study, excess deaths were concentrated in urban areas during the first wave of the pandemic but affected both urban and rural areas in the second wave [49].

A previous review and meta-analysis of global excess mortality reported a slightly higher estimate of excess mortality for lower-middle-income countries [133.45 (95% CI: 75.10–189.38) per 100,000]. Also, according to the COVID-19 Excess Mortality Collaborators, globally, the number of excess deaths due to the COVID-19 pandemic was largest in the regions of South Asia, north Africa the Middle East, and Eastern Europe. India (4·07 million [3·71–4·36]), the USA (1·13 million [1·08–1·18]), Russia (1·07 million [1·06–1·08]), Mexico (798,000 [741000–867000]), Brazil (792,000 [730000–847000]), Indonesia (736,000 [594000–955000]), and Pakistan (664,000 [498000–847000]) were estimated to have the highest cumulative excess deaths due to COVID-19 at the national level. They highlighted that across countries, the ratios showed significant variation, with New Zealand having the lowest at -17.10 (-26.06 to -8.84) and the Central African Republic the highest at 139.24 (88.86–213.67). South Africa, the only sub-Saharan African nation with available direct estimates of excess mortality from vital registration data, had a ratio of 3.31 (3.15–3.64). In South Asia, national-level ratios ranged from 8.33 (7.58–8.92) in India to 36.06 (15.14–53.25) in Bhutan. Within India and Pakistan, the most extreme ratios were observed at the state and province level, spanning from 0.96 (0.44–1.41) in Goa, India to 49.64 (28.94–72.74) in Balochistan, Pakistan [50].

By examining the methods employed in estimating excess mortality, we provide valuable insights into the diverse approaches used in LLMIC contexts. Notably, innovative techniques such as quantifying burial sites and utilizing geospatial analysis emerged during the pandemic, offering alternative means of mortality surveillance in resource-constrained settings. The methods of studies included in this review align with the methods of other studies conducted in high-income countries. ^50−^ Retrospective data analysis, while essential for calculating excess mortality, can be limited by delays in death registration, leading to potential underestimation at the time of analysis. This design was however suitable at the time of the pandemic and further corresponded to WHO recommendations.^53^ Estimating excess mortality requires an estimate of a certain level of baseline mortality to enable computation of excess mortality. Quantification of burial sites using geospatial analysis is a new method that emerged during the pandemic and was found to have considerable advantages for rapidly monitoring population mortality in settings without effective vital registrations [25]. However, this method could result in underestimation due to moderate precision because of missing grave counts in satellite images [26].

A few studies used burial site expansion before and after the pandemic to quantify excess mortality.

Some studies from the review used a combination of two or more methods, ranging from death registries, burial ground quantifications, journal reports and demographic survey data. The use of multiple methods is not new. It has been used in other studies [32, 51]. In this current review, linear regression models were widely used to estimate the number of deaths that would have occurred in the absence of the pandemic. This aligns with other estimation methods proven to be statistically efficient in estimating excess mortality [34].

There is relatively limited information on factors that influence excess mortality in LLMICs. Only one study included in our review [52] reported that excess mortality was associated with sociodemographic and clinical characteristics. [34], whereas in several high-income countries, socioeconomic disparity in excess mortality has been studied extensively. In England for example, it was observed that excess mortality was consistently higher for essential workers throughout 2020, particularly for healthcare workers [39]. In Korea, the pandemic has disproportionately affected those of lower socioeconomic status and has exacerbated inequalities in mortality [37]. Unfortunately, similar evidence is unavailable for LLMICs.

In this study, it is evident that the overall estimate is greatly influenced by the data from India due to its significant population size, constituting 65% of the weight. Consequently, the observed excess mortality rates in other countries appear considerably lower. This substantial variance could potentially be attributed to this influential factor for the high rates of excess mortality in LLMICs. It is plausible to speculate that excess mortality has been impacted by a wide range of factors, including limited health sector capacities to detect and treat COVID-19, more constrained resources to take care of other diseases, and fewer resources to cushion the negative social consequences of the pandemic [14].

The findings of this review reconfirm that the true impact of the pandemic is considerably higher than the reported number of COVID-19 deaths, which have been estimated at 100.3 /100,000 for the 10 LLMICs covered by studies included in our meta-analysis. Overall, our review shows the importance of addressing excess mortality in LLMICs and provides a foundation for ongoing research and policy initiatives aimed at improving pandemic preparedness and response strategies in these settings.

Our review has some limitations. First, a low number of primary studies met the criteria for inclusion and large variation in methods of included studies limited our ability to include studies in the meta-analysis. Second, our results are not representative of all LLMICs given insufficient numbers of studies from some parts of the world. Nevertheless, the results of this study provide a better understanding of the effect of the pandemic on mortality in LLMICs and may inform future analyses of excess mortality. The need to enhance death registration systems in LLMICs is essential for better pandemic monitoring.

## Conclusion

Our review shows that excess mortality during the COVID-19 pandemic was substantial in LLMICs. It was above excess mortality levels reported for HIC and much higher than reported COVID-19 deaths in LLMIC. Most studies used retrospective and linear regression models to estimate excess mortality. More research and better data are needed to identify the drivers of excess mortality in LLMICs.

### Supplementary Information

Supplementary Material 1.

Supplementary Material 2.

Supplementary Material 3.

## Data Availability

No datasets were generated or analysed during the current study.

## Abbreviations

ACM: All-Cause Mortality
BUA: Berlin University Alliance
COVID-19: Coronavirus Disease 2019
G-WAC: German-West African Centre for Global Health and Pandemic Prevention
HICs: High-Income Countries
LIC: Low-Income Countries
LMICs: Lower-Middle-Income Countries
LLMICs: Low- and Lower-Middle-Income Countries
NOS: Newcastle–Ottawa Scale
PRISMA: Preferred Reporting Items for Systematic Review and Meta-Analysis
PICO: Participants, Intervention, Comparator, and Outcome
WHO: World Health Organization
