# Supplementary material for: Excess mortality during the COVID-19 pandemic in low-and lower-middle-income countries: a systematic review and meta-analysis
Source: BMC Public Health. 2024 Jun 20;24:1643. doi: 10.1186/s12889-024-19154-w (PMC11188207; doi:10.1186/s12889-024-19154-w)
Supplement: Supplementary file 1 — Supplementary Material 1. [file 12889_2024_19154_MOESM1_ESM.docx]

**Appendix 1:** Quality assessment of cross-sectional studies

| **Risk of bias item** | **Hedstrom et al.** | **Besson et al.** | **Hanifi et al.** | **Gohari et al.,** | **Jha et al.** | **Tadbiri et al.** | **Ghafari et al.,** | **Otiende et al.** | **Watson et al.** | **Safavi‑Naini et al.** | **Lewnard et al.** | **Ghafari et al.** | **Rasambainarivo et al.** | **Wijaya** | **Leffler et al.** | **Barnwal et al.** | **Warsame et al.** | **Acosta et al.** | **Elyazar et al.** | **Rolando et al.** | **Esmaeilzadeh et al** | **Oduor et al.** | **Ebrahimoghli et al** | **Rabarison et al** |
| --- | --- | --- | --- | --- | --- | --- | --- | --- | --- | --- | --- | --- | --- | --- | --- | --- | --- | --- | --- | --- | --- | --- | --- | --- |
| Was the study’s target population a close representation of the national population in relation to relevant variables, e.g. age, sex, occupation | Yes | Yes | Yes | Yes | Yes | Yes | Yes | Yes | Yes | Yes | Yes | Yes | Yes | Yes | Yes | Yes | Yes | Yes | Yes | Yes | Yes | Yes | Yes | Yes |
| Was the sampling frame a true or close representation of the target population? | Yes | Yes | Yes | Yes | Yes | Yes | Yes | Yes | Yes | Yes | Yes | Yes | Yes | Yes | Yes | Yes | Yes | Yes | Yes | Yes | Yes | Yes | Yes | Yes |
| Was some form of random selection used to select the sample, OR, was a census undertaken? | No | No | No | No | No | No | No | No | No | No | No | No | No | No | No | No | No | No | No | No | No | No | No | No |
| Was the likelihood of non-response bias minimal? | Yes | Yes | Yes | Yes | Yes | Yes | Yes | Yes | Yes | Yes | Yes | Yes | Yes | Yes | Yes | Yes | Yes | Yes | Yes | Yes | Yes | Yes | Yes | Yes |
| Were data collected directly from the subjects (as opposed to a proxy)? | No | No | No | No | No | No | No | No | No | No | No | No | No | No | No | No | No | No | No | No | No | No | No | No |
| Was an acceptable case definition used in the study? | Yes | Yes | Yes | Yes | Yes | Yes | Yes | Yes | Yes | Yes | Yes | Yes | Yes | Yes | Yes | Yes | Yes | Yes | Yes | Yes | Yes | Yes | Yes | Yes |
| Was the study instrument that measured the parameter of interest shown to have reliability and validity | Yes | Yes | Yes | Yes | Yes | Yes | Yes | Yes | Yes | Yes | Yes | Yes | Yes | Yes | Yes | Yes | Yes | Yes | Yes | Yes | Yes | Yes | Yes | Yes |
| Was the same mode of data collection used for all subjects? | Yes | Yes | Yes | Yes | Yes | Yes | Yes | Yes | Yes | Yes | Yes | Yes | Yes | Yes | Yes | Yes | Yes | Yes | Yes | Yes | Yes | Yes | Yes | Yes |
| Was the length of the shortest prevalence period for the parameter of interest appropriate? | Yes | Yes | Yes | Yes | Yes | Yes | Yes | Yes | Yes | Yes | Yes | Yes | Yes | Yes | Yes | Yes | Yes | Yes | Yes | Yes | Yes | Yes | Yes | Yes |
| Were the numerator(s) and denominator(s) for the parameter of interest appropriate? | Yes | Yes | Yes | Yes | Yes | Yes | Yes | Yes | Yes | Yes | Yes | Yes | Yes | Yes | Yes | Yes | Yes | Yes | Yes | Yes | Yes | Yes | Yes | Yes |
| Risk of bias | Low | Low | Low | Low | Low | Low | Low | Low | Low | Low | Low | Low | Low | Low | Low | Low | Low | Low | Low | Low | Low | Low | Low | Low |
