# Supplementary material for: Excess mortality during the COVID-19 pandemic in low-and lower-middle-income countries: a systematic review and meta-analysis
Source: BMC Public Health. 2024 Jun 20;24:1643. doi: 10.1186/s12889-024-19154-w (PMC11188207; doi:10.1186/s12889-024-19154-w)
Supplement: Supplementary file 2 — Supplementary Material 2. [file 12889_2024_19154_MOESM2_ESM.docx]

**Search Strategies**

#**Web of Science Search Strategy (v0.1)**

1: (ALL=((COVID-19 [Mesh]) OR (COVID-19) OR (SARS-CoV-2) OR (Severe Acute Respiratory Syndrome Coronavirus 2) OR (COVID-19 pandemic) OR (Pandemic) OR (COVID*) OR (Coronaviru*) OR (SARS Coronavirus*) OR (SARS-COV*))) AND ALL=((Mortality [Mesh]) OR (Excess mortalit*) OR (All-cause mortality) OR (excess death*) OR (Additional death*) OR (Increase mortalit*) OR (higher mortality) OR (Excess fatalit*))

2: ALL=( (Developing Countries [Mesh]) OR (Least developed countries) OR (LDC) OR (Developing Countries) OR (Low-income countries) OR (LIC) OR (Lower- Middle-income countries) OR (LMC) OR (Low-and-lower-middle income countries) OR (LLMIC) OR (Afghanistan) OR (Algeria) OR (Angola) OR (Bangladesh) OR (Benin) OR (Bhutan) OR (Bolivia))

3: ALL=((Burkina Faso) OR (Burundi) OR (Cabo Verde) OR (Cambodia) OR (Cameroon) OR (Central African Republic) OR (C.A.R) OR (Chad) OR (Comoros) OR (Democratic Republic of the Congo) OR (DRC) OR (Democratic Republic Congo) OR (Republic of the Congo) OR (Costa Rica) OR (Cote d'Ivoire) OR (Côte d'Ivoire) OR (Ivory Coast) OR (Djibouti) )

4: ALL=((Arab Republic of Egypt) OR (El Salvador) OR (Republic El Salvador) OR (Eritrea) OR (Eswatini) OR (Ethiopia) OR (Gambia) OR (The Gambia) OR (Ghana) OR (Guinea) OR

(Guinea-Bissau) OR (Haiti) OR (Honduras) OR (India) OR (Indonesia) OR (Islamic Republic of

Iran))

5: ALL=((Iran) OR (Persia) OR (Kenya) OR (Kiribati) OR (democratic People's Republic of Korea) OR (Kyrgyz republic) OR (Kyrgyzstan) OR (Laos) OR (Lao People's Democratic Republic) or (LAO PDR) OR (Lebanon) OR (Lesotho) OR (Liberia) OR(Madagascar) OR

(Malawi) OR (Mali) OR (Mauritania) OR (Federated States of Micronesia))

6: ALL=((Micronesia) OR (Mongolia) OR (Morocco) OR (Mozambique) OR (Myanmar) OR (Nepal) OR (Nicaragua) OR (Niger) OR (Nigeria) OR (Pakistan) OR (Papua New Guinea) OR (PNG) OR (Philippines) OR (Rwanda) OR (Samoa) OR (São Tomé and Príncipe) OR (Sao Tome

and Principe) OR (Senegal) OR (Serbia) OR (Sierra Leone))

7: ALL=((Solomon islands) OR (Somalia) OR (South sudan) OR (Sri lanka) OR (Sudan) OR (Syria) OR (Syrian Arab republic) OR (Tajikistan) OR (Tanzania) OR (Timor-leste) OR (Togo) OR (Tunisia) OR (Uganda) OR (Ukraine) OR (Uzbekistan) OR (Vanuatu) OR (Vietnam) OR (West bank and gaza) OR (Yemeni Republic) OR (Republic of Yemen) OR (Yemen) OR (Zambia) OR (Zimbabwe) )

8: ALL=(((Mortality [Mesh]) OR (Excess mortalit*) OR (All-cause mortality) OR (excess death*)

OR (Additional death*) OR (Increase mortalit*) OR (higher mortality) OR (Excess

fatalit*)))

9: #2 AND #3 AND #4

10: #2 AND #3 AND #4

11: #2 AND #5 AND #6

12: #2 AND #7 AND #8

**#Embase**

| **Key concepts** | **Search details** |
| --- | --- |
| **COVID-19** | 'covid 19' OR 'sars cov 2' OR (severe AND acute AND respiratory AND syndrome AND coronavirus AND 2) OR ('covid 19' AND pandemic) OR pandemic OR covid* OR coronaviru* OR (sars AND coronavirus*) OR 'sars cov*' |
| **Excess Mortality** | excess AND mortalit* OR ('all cause' AND mortality) OR (excess AND death*) OR (additional AND death*) OR (increase AND mortalit*) OR (higher AND mortality) OR (excess AND fatalit*) |
| **Low -and lower-middle income countries** | 'low and middle-income countries' OR 'least developed countries' OR 'developing countries'/exp OR 'developing countries' OR 'afghanistan'/exp OR 'afghanistan' OR 'algeria'/exp OR 'algeria' OR 'angola'/exp OR 'angola' OR 'ldc' OR 'lic' OR 'lmc' OR 'llmic' OR 'bangladesh'/exp OR 'bangladesh' OR 'benin'/exp OR 'benin' OR 'bhutan'/exp OR 'bhutan' OR 'bolivia'/exp OR 'bolivia' OR 'burkina faso'/exp OR 'burkina faso' OR 'burundi'/exp OR 'burundi' OR 'cabo verde'/exp OR 'cabo verde' OR 'cambodia'/exp OR 'cambodia' OR 'cameroon'/exp OR 'cameroon' OR 'central african republic'/exp OR 'central african republic' OR 'chad'/exp OR 'chad' OR 'comoros'/exp OR 'comoros' OR 'democratic republic of the congo'/exp OR 'democratic republic of the congo' OR 'republic of the congo' OR 'costa rica'/exp OR 'costa rica' OR 'ivory coast'/exp OR 'ivory coast' OR 'djibouti'/exp OR 'djibouti' OR 'arab republic of egypt' OR 'el salvador'/exp OR 'el salvador' OR 'eritrea'/exp OR 'eritrea' OR 'eswatini'/exp OR 'eswatini' OR 'ethiopia'/exp OR 'ethiopia' OR 'gambia'/exp OR 'gambia' OR 'ghana'/exp OR 'ghana' OR 'guinea'/exp OR 'guinea' OR 'guinea-bissau' OR 'haiti'/exp OR 'haiti' OR 'honduras'/exp OR 'honduras' OR 'india'/exp OR 'india' OR 'indonesia'/exp OR 'indonesia' OR 'islamic republic of iran'/exp OR 'islamic republic of iran' OR 'kenya'/exp OR 'kenya' OR 'kiribati'/exp OR 'kiribati' OR 'the democratic peoples republic of korea' OR 'kyrgyzstan' OR 'kyrgyz republic'/exp OR 'kyrgyz republic' OR 'lao peoples democratic republic' OR 'lebanon'/exp OR 'lebanon' OR 'lesotho'/exp OR 'lesotho' OR 'liberia'/exp OR 'liberia' OR 'madagascar'/exp OR 'madagascar' OR 'malawi'/exp OR 'malawi' OR 'mali'/exp OR 'mali' OR 'mauritania'/exp OR 'mauritania' OR 'persia' OR 'federated states of micronesia'/exp OR 'micronesia' OR 'mongolia'/exp OR 'mongolia' OR 'morocco'/exp OR 'morocco' OR 'mozambique'/exp OR 'mozambique' OR 'myanmar'/exp OR 'myanmar' OR 'nepal'/exp OR 'nepal' OR 'nicaragua'/exp OR 'nicaragua' OR 'niger'/exp OR 'niger' OR 'nigeria'/exp OR 'nigeria' OR 'pakistan'/exp OR 'pakistan' OR 'papua new guinea'/exp OR 'papua new guinea' OR 'philippines'/exp OR 'philippines' OR 'rwanda'/exp OR 'rwanda' OR 'samoa'/exp OR 'samoa' OR 'são tomé and príncipe' OR 'senegal'/exp OR 'senegal' OR 'serbia'/exp OR 'serbia' OR 'sierra leone'/exp OR 'sierra leone' OR 'solomon islands'/exp OR 'solomon islands' OR 'somalia'/exp OR 'somalia' OR 'south sudan'/exp OR 'south sudan' OR 'sri lanka'/exp OR 'sri lanka' OR 'st. lucia'/exp OR 'st. lucia' OR 'sudan'/exp OR 'sudan' OR 'syrian arab republic'/exp OR 'syria' OR 'syrian arab republic' OR 'tajikistan'/exp OR 'tajikistan' OR 'tanzania'/exp OR 'tanzania' OR 'timor-leste'/exp OR 'timor-leste' OR 'togo'/exp OR 'togo' OR 'tunisia'/exp OR 'tunisia' OR 'uganda'/exp OR 'uganda' OR 'ukraine'/exp OR 'ukraine' OR 'uzbekistan'/exp OR 'uzbekistan' OR 'vanuatu'/exp OR 'vanuatu' OR 'vietnam'/exp OR 'vietnam' OR 'west bank and gaza' OR 'republic of yemen'/exp OR 'republic of yemen' OR 'zambia'/exp OR 'zambia' OR 'zimbabwe'/exp OR 'zimbabwe' |
| **COMBINED KEY CONCEPTS** | ('covid 19' OR 'sars cov 2' OR (severe AND acute AND respiratory AND syndrome AND coronavirus AND 2) OR ('covid 19' AND pandemic) OR pandemic OR covid* OR coronaviru* OR (sars AND coronavirus*) OR 'sars cov*') AND (excess AND mortalit* OR ('all cause' AND mortality) OR (excess AND death*) OR (additional AND death*) OR (increase AND mortalit*) OR (higher AND mortality) OR (excess AND fatalit*)) AND ('low and middle-income countries' OR 'least developed countries' OR 'developing countries'/exp OR 'developing countries' OR 'afghanistan'/exp OR 'afghanistan' OR 'algeria'/exp OR 'algeria' OR 'angola'/exp OR 'angola' OR 'ldc' OR 'lic' OR 'lmc' OR 'llmic' OR 'bangladesh'/exp OR 'bangladesh' OR 'benin'/exp OR 'benin' OR 'bhutan'/exp OR 'bhutan' OR 'bolivia'/exp OR 'bolivia' OR 'burkina faso'/exp OR 'burkina faso' OR 'burundi'/exp OR 'burundi' OR 'cabo verde'/exp OR 'cabo verde' OR 'cambodia'/exp OR 'cambodia' OR 'cameroon'/exp OR 'cameroon' OR 'central african republic'/exp OR 'central african republic' OR 'chad'/exp OR 'chad' OR 'comoros'/exp OR 'comoros' OR 'democratic republic of the congo'/exp OR 'democratic republic of the congo' OR 'republic of the congo' OR 'costa rica'/exp OR 'costa rica' OR 'ivory coast'/exp OR 'ivory coast' OR 'djibouti'/exp OR 'djibouti' OR 'arab republic of egypt' OR 'el salvador'/exp OR 'el salvador' OR 'eritrea'/exp OR 'eritrea' OR 'eswatini'/exp OR 'eswatini' OR 'ethiopia'/exp OR 'ethiopia' OR 'gambia'/exp OR 'gambia' OR 'ghana'/exp OR 'ghana' OR 'guinea'/exp OR 'guinea' OR 'guinea-bissau' OR 'haiti'/exp OR 'haiti' OR 'honduras'/exp OR 'honduras' OR 'india'/exp OR 'india' OR 'indonesia'/exp OR 'indonesia' OR 'islamic republic of iran'/exp OR 'islamic republic of iran' OR 'kenya'/exp OR 'kenya' OR 'kiribati'/exp OR 'kiribati' OR 'the democratic peoples republic of korea' OR 'kyrgyzstan' OR 'kyrgyz republic'/exp OR 'kyrgyz republic' OR 'lao peoples democratic republic' OR 'lebanon'/exp OR 'lebanon' OR 'lesotho'/exp OR 'lesotho' OR 'liberia'/exp OR 'liberia' OR 'madagascar'/exp OR 'madagascar' OR 'malawi'/exp OR 'malawi' OR 'mali'/exp OR 'mali' OR 'mauritania'/exp OR 'mauritania' OR 'persia' OR 'federated states of micronesia'/exp OR 'micronesia' OR 'mongolia'/exp OR 'mongolia' OR 'morocco'/exp OR 'morocco' OR 'mozambique'/exp OR 'mozambique' OR 'myanmar'/exp OR 'myanmar' OR 'nepal'/exp OR 'nepal' OR 'nicaragua'/exp OR 'nicaragua' OR 'niger'/exp OR 'niger' OR 'nigeria'/exp OR 'nigeria' OR 'pakistan'/exp OR 'pakistan' OR 'papua new guinea'/exp OR 'papua new guinea' OR 'philippines'/exp OR 'philippines' OR 'rwanda'/exp OR 'rwanda' OR 'samoa'/exp OR 'samoa' OR 'são tomé and príncipe' OR 'senegal'/exp OR 'senegal' OR 'serbia'/exp OR 'serbia' OR 'sierra leone'/exp OR 'sierra leone' OR 'solomon islands'/exp OR 'solomon islands' OR 'somalia'/exp OR 'somalia' OR 'south sudan'/exp OR 'south sudan' OR 'sri lanka'/exp OR 'sri lanka' OR 'st. lucia'/exp OR 'st. lucia' OR 'sudan'/exp OR 'sudan' OR 'syrian arab republic'/exp OR 'syria' OR 'syrian arab republic' OR 'tajikistan'/exp OR 'tajikistan' OR 'tanzania'/exp OR 'tanzania' OR 'timor-leste'/exp OR 'timor-leste' OR 'togo'/exp OR 'togo' OR 'tunisia'/exp OR 'tunisia' OR 'uganda'/exp OR 'uganda' OR 'ukraine'/exp OR 'ukraine' OR 'uzbekistan'/exp OR 'uzbekistan' OR 'vanuatu'/exp OR 'vanuatu' OR 'vietnam'/exp OR 'vietnam' OR 'west bank and gaza' OR 'republic of yemen'/exp OR 'republic of yemen' OR 'zambia'/exp OR 'zambia' OR 'zimbabwe'/exp OR 'zimbabwe') AND [abstracts]/lim |

**#PubMed**

| **Key concept** | **Search details** |
| --- | --- |
| **COVID-19** | "covid 19"[MeSH Terms] OR ("covid 19"[All Fields] OR "covid 19"[MeSH Terms] OR "covid 19 vaccines"[All Fields] OR "covid 19 vaccines"[MeSH Terms] OR "covid 19 serotherapy"[All Fields] OR "covid 19 serotherapy"[Supplementary Concept] OR "covid 19 nucleic acid testing"[All Fields] OR "covid 19 nucleic acid testing"[MeSH Terms] OR "covid 19 serological testing"[All Fields] OR "covid 19 serological testing"[MeSH Terms] OR "covid 19 testing"[All Fields] OR "covid 19 testing"[MeSH Terms] OR "sars cov 2"[All Fields] OR "sars cov 2"[MeSH Terms] OR "severe acute respiratory syndrome coronavirus 2"[All Fields] OR "ncov"[All Fields] OR "2019 ncov"[All Fields] OR (("coronavirus"[MeSH Terms] OR "coronavirus"[All Fields] OR "cov"[All Fields]) AND 2019/11/01:3000/12/31[Date - Publication])) OR ("sars cov 2"[MeSH Terms] OR "sars cov 2"[All Fields] OR "sars cov 2"[All Fields]) OR ("sars cov 2"[MeSH Terms] OR "sars cov 2"[All Fields] OR "severe acute respiratory syndrome coronavirus 2"[All Fields]) OR ("covid 19"[MeSH Terms] OR "covid 19"[All Fields] OR "covid 19 pandemic"[All Fields]) OR ("pandemic s"[All Fields] OR "pandemically"[All Fields] OR "pandemicity"[All Fields] OR "pandemics"[MeSH Terms] OR "pandemics"[All Fields] OR "pandemic"[All Fields]) OR "covid*"[All Fields] OR "coronaviru*"[All Fields] OR ("SARS"[All Fields] AND "coronavirus*"[All Fields]) OR "sars cov*"[All Fields] |
| **Excess Mortality** | "mortality"[MeSH Terms] OR (("excess"[All Fields] OR "excesses"[All Fields] OR "excessive"[All Fields] OR "excessively"[All Fields]) AND "mortalit*"[All Fields]) OR ("All-cause"[All Fields] AND ("mortality"[MeSH Terms] OR "mortality"[All Fields] OR "mortalities"[All Fields] OR "mortality"[MeSH Subheading])) OR (("excess"[All Fields] OR "excesses"[All Fields] OR "excessive"[All Fields] OR "excessively"[All Fields]) AND "death*"[All Fields]) OR (("addition"[All Fields] OR "additional"[All Fields] OR "additions"[All Fields] OR "additive"[All Fields] OR "additively"[All Fields] OR "additives"[All Fields] OR "additivities"[All Fields] OR "additivity"[All Fields]) AND "death*"[All Fields]) OR (("increase"[All Fields] OR "increased"[All Fields] OR "increases"[All Fields] OR "increasing"[All Fields] OR "increasings"[All Fields]) AND "mortalit*"[All Fields]) OR ("higher"[All Fields] AND ("mortality"[MeSH Terms] OR "mortality"[All Fields] OR "mortalities"[All Fields] OR "mortality"[MeSH Subheading])) OR (("excess"[All Fields] OR "excesses"[All Fields] OR "excessive"[All Fields] OR "excessively"[All Fields]) AND "fatalit*"[All Fields]) |
| **Low-and-lower -middle income countries** | "developing countries"[MeSH Terms] OR ("developing countries"[MeSH Terms] OR ("developing"[All Fields] AND "countries"[All Fields]) OR "developing countries"[All Fields] OR ("least"[All Fields] AND "developed"[All Fields] AND "countries"[All Fields]) OR "least developed countries"[All Fields]) OR "LDC"[All Fields] OR ("developing countries"[MeSH Terms] OR ("developing"[All Fields] AND "countries"[All Fields]) OR "developing countries"[All Fields]) OR (("poverty"[MeSH Terms] OR "poverty"[All Fields] OR ("low"[All Fields] AND "income"[All Fields]) OR "low income"[All Fields]) AND ("countries"[All Fields] OR "country"[All Fields] OR "country s"[All Fields] OR "countrys"[All Fields])) OR "LIC"[All Fields] OR (("lower"[All Fields] OR "lowered"[All Fields] OR "lowering"[All Fields] OR "lowerings"[All Fields] OR "lowers"[All Fields]) AND "Middle-income"[All Fields] AND ("countries"[All Fields] OR "country"[All Fields] OR "country s"[All Fields] OR "countrys"[All Fields])) OR "LMC"[All Fields] OR ("Low-and-lower-middle"[All Fields] AND ("income"[MeSH Terms] OR "income"[All Fields] OR "incomes"[All Fields] OR "income s"[All Fields]) AND ("countries"[All Fields] OR "country"[All Fields] OR "country s"[All Fields] OR "countrys"[All Fields])) OR ("llmic"[All Fields] OR "llmics"[All Fields]) OR ("afghanistan"[MeSH Terms] OR "afghanistan"[All Fields] OR "afghanistan s"[All Fields]) OR ("algeria"[MeSH Terms] OR "algeria"[All Fields]) OR ("angola"[MeSH Terms] OR "angola"[All Fields] OR "angola s"[All Fields]) OR ("bangladesh"[MeSH Terms] OR "bangladesh"[All Fields] OR "bangladesh s"[All Fields]) OR ("benin"[MeSH Terms] OR "benin"[All Fields] OR "benin s"[All Fields]) OR ("bhutan"[MeSH Terms] OR "bhutan"[All Fields] OR "bhutan s"[All Fields]) OR ("bolivia"[MeSH Terms] OR "bolivia"[All Fields]) OR ("burkina faso"[MeSH Terms] OR ("burkina"[All Fields] AND "faso"[All Fields]) OR "burkina faso"[All Fields]) OR ("burundi"[MeSH Terms] OR "burundi"[All Fields]) OR ("cabo verde"[MeSH Terms] OR ("cabo"[All Fields] AND "verde"[All Fields]) OR "cabo verde"[All Fields]) OR ("cambodia"[MeSH Terms] OR "cambodia"[All Fields] OR "cambodia s"[All Fields]) OR ("cameroon"[MeSH Terms] OR "cameroon"[All Fields] OR "cameroons"[All Fields] OR "cameroon s"[All Fields]) OR ("central african republic"[MeSH Terms] OR ("central"[All Fields] AND "african"[All Fields] AND "republic"[All Fields]) OR "central african republic"[All Fields]) OR "C.A.R"[All Fields] OR ("chad"[MeSH Terms] OR "chad"[All Fields]) OR ("comoros"[MeSH Terms] OR "comoros"[All Fields] OR "comoro"[All Fields]) OR ("democratic republic of the congo"[MeSH Terms] OR ("democratic"[All Fields] AND "republic"[All Fields] AND "congo"[All Fields]) OR "democratic republic of the congo"[All Fields]) OR "DRC"[All Fields] OR (("democrat"[All Fields] OR "democratic"[All Fields] OR "democratically"[All Fields] OR "democratization"[All Fields] OR "democratize"[All Fields] OR "democratized"[All Fields] OR "democratizing"[All Fields] OR "democrats"[All Fields]) AND ("republic"[All Fields] OR "republic s"[All Fields] OR "republics"[All Fields]) AND ("congo"[MeSH Terms] OR "congo"[All Fields])) OR ("congo"[MeSH Terms] OR "congo"[All Fields] OR ("republic"[All Fields] AND "congo"[All Fields]) OR "republic of the congo"[All Fields]) OR ("costa rica"[MeSH Terms] OR ("costa"[All Fields] AND "rica"[All Fields]) OR "costa rica"[All Fields]) OR ("cote d ivoire"[MeSH Terms] OR ("cote"[All Fields] AND "d ivoire"[All Fields]) OR "cote d ivoire"[All Fields]) OR ("cote d ivoire"[MeSH Terms] OR ("cote"[All Fields] AND "d ivoire"[All Fields]) OR "cote d ivoire"[All Fields]) OR ("cote d ivoire"[MeSH Terms] OR ("cote"[All Fields] AND "d ivoire"[All Fields]) OR "cote d ivoire"[All Fields] OR ("ivory"[All Fields] AND "coast"[All Fields]) OR "ivory coast"[All Fields]) OR ("djibouti"[MeSH Terms] OR "djibouti"[All Fields]) OR ("egypt"[MeSH Terms] OR "egypt"[All Fields] OR ("arab"[All Fields] AND "republic"[All Fields] AND "egypt"[All Fields]) OR "arab republic of egypt"[All Fields]) OR ("el salvador"[MeSH Terms] OR ("el"[All Fields] AND "salvador"[All Fields]) OR "el salvador"[All Fields]) OR (("republic"[All Fields] OR "republic s"[All Fields] OR "republics"[All Fields]) AND ("el salvador"[MeSH Terms] OR ("el"[All Fields] AND "salvador"[All Fields]) OR "el salvador"[All Fields])) OR ("eritrea"[MeSH Terms] OR "eritrea"[All Fields]) OR ("eswatini"[MeSH Terms] OR "eswatini"[All Fields]) OR ("ethiopia"[MeSH Terms] OR "ethiopia"[All Fields] OR "ethiopia s"[All Fields]) OR ("gambia"[MeSH Terms] OR "gambia"[All Fields] OR "gambia s"[All Fields]) OR ("gambia"[MeSH Terms] OR "gambia"[All Fields] OR "the gambia"[All Fields]) OR ("ghana"[MeSH Terms] OR "ghana"[All Fields] OR "ghana s"[All Fields]) OR ("guinea"[MeSH Terms] OR "guinea"[All Fields] OR "guinea s"[All Fields] OR "guineas"[All Fields]) OR ("guinea bissau"[MeSH Terms] OR "guinea bissau"[All Fields] OR ("guinea"[All Fields] AND "bissau"[All Fields]) OR "guinea bissau"[All Fields]) OR ("haiti"[MeSH Terms] OR "haiti"[All Fields] OR "haiti s"[All Fields]) OR ("honduras"[MeSH Terms] OR "honduras"[All Fields]) OR ("india"[MeSH Terms] OR "india"[All Fields] OR "india s"[All Fields] OR "indias"[All Fields]) OR ("indonesia"[MeSH Terms] OR "indonesia"[All Fields] OR "indonesia s"[All Fields] OR "indonesias"[All Fields]) OR ("iran"[MeSH Terms] OR "iran"[All Fields] OR ("islamic"[All Fields] AND "republic"[All Fields] AND "iran"[All Fields]) OR "islamic republic of iran"[All Fields]) OR ("iran"[MeSH Terms] OR "iran"[All Fields]) OR ("persia"[MeSH Terms] OR "persia"[All Fields]) OR ("kenya"[MeSH Terms] OR "kenya"[All Fields] OR "kenya s"[All Fields]) OR ("micronesia"[MeSH Terms] OR "micronesia"[All Fields] OR "kiribati"[All Fields]) OR ("democratic people s republic of korea"[MeSH Terms] OR ("democratic"[All Fields] AND "people s"[All Fields] AND "republic"[All Fields] AND "korea"[All Fields]) OR "democratic people s republic of korea"[All Fields]) OR ("kyrgyzstan"[MeSH Terms] OR "kyrgyzstan"[All Fields] OR ("kyrgyz"[All Fields] AND "republic"[All Fields]) OR "kyrgyz republic"[All Fields]) OR ("kyrgyzstan"[MeSH Terms] OR "kyrgyzstan"[All Fields]) OR ("laos"[MeSH Terms] OR "laos"[All Fields]) OR ("laos"[MeSH Terms] OR "laos"[All Fields] OR ("LAO"[All Fields] AND "people s"[All Fields] AND "democratic"[All Fields] AND "republic"[All Fields]) OR "lao people s democratic republic"[All Fields]) OR ("LAO"[All Fields] AND "PDR"[All Fields]) OR ("lebanon"[MeSH Terms] OR "lebanon"[All Fields] OR "lebanon s"[All Fields]) OR ("lesotho"[MeSH Terms] OR "lesotho"[All Fields]) OR ("liberia"[MeSH Terms] OR "liberia"[All Fields] OR "liberia s"[All Fields]) OR ("madagascar"[MeSH Terms] OR "madagascar"[All Fields] OR "madagascar s"[All Fields]) OR ("malawi"[MeSH Terms] OR "malawi"[All Fields] OR "malawi s"[All Fields]) OR ("mali"[MeSH Terms] OR "mali"[All Fields]) OR ("mauritania"[MeSH Terms] OR "mauritania"[All Fields]) OR ("micronesia"[MeSH Terms] OR "micronesia"[All Fields] OR ("federated"[All Fields] AND "states"[All Fields] AND "micronesia"[All Fields]) OR "federated states of micronesia"[All Fields]) OR ("micronesia"[MeSH Terms] OR "micronesia"[All Fields]) OR ("mongolia"[MeSH Terms] OR "mongolia"[All Fields] OR "mongolia s"[All Fields]) OR ("morocco"[MeSH Terms] OR "morocco"[All Fields]) OR ("mozambique"[MeSH Terms] OR "mozambique"[All Fields] OR "mozambique s"[All Fields]) OR ("myanmar"[MeSH Terms] OR "myanmar"[All Fields] OR "myanmar s"[All Fields] OR "myanmars"[All Fields]) OR ("nepal"[MeSH Terms] OR "nepal"[All Fields] OR "nepal s"[All Fields]) OR ("nicaragua"[MeSH Terms] OR "nicaragua"[All Fields] OR "nicaragua s"[All Fields]) OR ("niger"[MeSH Terms] OR "niger"[All Fields]) OR ("nigeria"[MeSH Terms] OR "nigeria"[All Fields] OR "nigeria s"[All Fields]) OR ("pakistan"[MeSH Terms] OR "pakistan"[All Fields] OR "pakistan s"[All Fields]) OR ("papua new guinea"[MeSH Terms] OR ("papua"[All Fields] AND "new"[All Fields] AND "guinea"[All Fields]) OR "papua new guinea"[All Fields]) OR "PNG"[All Fields] OR ("philippine"[All Fields] OR "philippines"[MeSH Terms] OR "philippines"[All Fields]) OR ("rwanda"[MeSH Terms] OR "rwanda"[All Fields] OR "rwanda s"[All Fields]) OR ("samoa"[MeSH Terms] OR "samoa"[All Fields] OR "samoas"[All Fields]) OR ("sao tome and principe"[MeSH Terms] OR ("sao"[All Fields] AND "tome"[All Fields] AND "principe"[All Fields]) OR "sao tome and principe"[All Fields]) OR ("sao tome and principe"[MeSH Terms] OR ("sao"[All Fields] AND "tome"[All Fields] AND "principe"[All Fields]) OR "sao tome and principe"[All Fields]) OR ("senegal"[MeSH Terms] OR "senegal"[All Fields] OR "senegal s"[All Fields]) OR ("serbia"[MeSH Terms] OR "serbia"[All Fields]) OR ("sierra leone"[MeSH Terms] OR ("sierra"[All Fields] AND "leone"[All Fields]) OR "sierra leone"[All Fields]) OR ("melanesia"[MeSH Terms] OR "melanesia"[All Fields] OR ("solomon"[All Fields] AND "islands"[All Fields]) OR "solomon islands"[All Fields]) OR ("somalia"[MeSH Terms] OR "somalia"[All Fields]) OR ("south sudan"[MeSH Terms] OR ("south"[All Fields] AND "sudan"[All Fields]) OR "south sudan"[All Fields]) OR ("sri lanka"[MeSH Terms] OR ("sri"[All Fields] AND "lanka"[All Fields]) OR "sri lanka"[All Fields]) OR ("sudan"[MeSH Terms] OR "sudan"[All Fields] OR "sudans"[All Fields] OR "sudan s"[All Fields]) OR ("syria"[MeSH Terms] OR "syria"[All Fields] OR "syria s"[All Fields]) OR ("syria"[MeSH Terms] OR "syria"[All Fields] OR ("syrian"[All Fields] AND "arab"[All Fields] AND "republic"[All Fields]) OR "syrian arab republic"[All Fields]) OR ("tajikistan"[MeSH Terms] OR "tajikistan"[All Fields]) OR ("tanzania"[MeSH Terms] OR "tanzania"[All Fields] OR "tanzania s"[All Fields]) OR ("timor leste"[MeSH Terms] OR "timor leste"[All Fields] OR ("timor"[All Fields] AND "leste"[All Fields]) OR "timor leste"[All Fields]) OR ("togo"[MeSH Terms] OR "togo"[All Fields]) OR ("tunisia"[MeSH Terms] OR "tunisia"[All Fields]) OR ("uganda"[MeSH Terms] OR "uganda"[All Fields] OR "uganda s"[All Fields]) OR ("ukraine"[MeSH Terms] OR "ukraine"[All Fields] OR "ukraine s"[All Fields]) OR ("uzbekistan"[MeSH Terms] OR "uzbekistan"[All Fields]) OR ("vanuatu"[MeSH Terms] OR "vanuatu"[All Fields]) OR ("vietnam"[MeSH Terms] OR "vietnam"[All Fields] OR "vietnam s"[All Fields]) OR (("middle east"[MeSH Terms] OR ("middle"[All Fields] AND "east"[All Fields]) OR "middle east"[All Fields] OR ("west"[All Fields] AND "bank"[All Fields]) OR "west bank"[All Fields]) AND "gaza"[All Fields]) OR (("yemeni"[All Fields] OR "yemenis"[All Fields]) AND ("republic"[All Fields] OR "republic s"[All Fields] OR "republics"[All Fields])) OR ("yemen"[MeSH Terms] OR "yemen"[All Fields] OR ("republic"[All Fields] AND "yemen"[All Fields]) OR "republic of yemen"[All Fields]) OR ("yemen"[MeSH Terms] OR "yemen"[All Fields]) OR ("zambia"[MeSH Terms] OR "zambia"[All Fields] OR "zambia s"[All Fields]) OR ("zimbabwe"[MeSH Terms] OR "zimbabwe"[All Fields] OR "zimbabwe s"[All Fields]) |
| **ALL CONCEPTS COMBINED** | ("developing countries"[MeSH Terms] OR ("developing countries"[MeSH Terms] OR ("developing"[All Fields] AND "countries"[All Fields]) OR "developing countries"[All Fields] OR ("least"[All Fields] AND "developed"[All Fields] AND "countries"[All Fields]) OR "least developed countries"[All Fields]) OR "LDC"[All Fields] OR ("developing countries"[MeSH Terms] OR ("developing"[All Fields] AND "countries"[All Fields]) OR "developing countries"[All Fields]) OR (("poverty"[MeSH Terms] OR "poverty"[All Fields] OR ("low"[All Fields] AND "income"[All Fields]) OR "low income"[All Fields]) AND ("countries"[All Fields] OR "country"[All Fields] OR "country s"[All Fields] OR "countrys"[All Fields])) OR "LIC"[All Fields] OR (("lower"[All Fields] OR "lowered"[All Fields] OR "lowering"[All Fields] OR "lowerings"[All Fields] OR "lowers"[All Fields]) AND "Middle-income"[All Fields] AND ("countries"[All Fields] OR "country"[All Fields] OR "country s"[All Fields] OR "countrys"[All Fields])) OR "LMC"[All Fields] OR ("Low-and-lower-middle"[All Fields] AND ("income"[MeSH Terms] OR "income"[All Fields] OR "incomes"[All Fields] OR "income s"[All Fields]) AND ("countries"[All Fields] OR "country"[All Fields] OR "country s"[All Fields] OR "countrys"[All Fields])) OR ("llmic"[All Fields] OR "llmics"[All Fields]) OR ("afghanistan"[MeSH Terms] OR "afghanistan"[All Fields] OR "afghanistan s"[All Fields]) OR ("algeria"[MeSH Terms] OR "algeria"[All Fields]) OR ("angola"[MeSH Terms] OR "angola"[All Fields] OR "angola s"[All Fields]) OR ("bangladesh"[MeSH Terms] OR "bangladesh"[All Fields] OR "bangladesh s"[All Fields]) OR ("benin"[MeSH Terms] OR "benin"[All Fields] OR "benin s"[All Fields]) OR ("bhutan"[MeSH Terms] OR "bhutan"[All Fields] OR "bhutan s"[All Fields]) OR ("bolivia"[MeSH Terms] OR "bolivia"[All Fields]) OR ("burkina faso"[MeSH Terms] OR ("burkina"[All Fields] AND "faso"[All Fields]) OR "burkina faso"[All Fields]) OR ("burundi"[MeSH Terms] OR "burundi"[All Fields]) OR ("cabo verde"[MeSH Terms] OR ("cabo"[All Fields] AND "verde"[All Fields]) OR "cabo verde"[All Fields]) OR ("cambodia"[MeSH Terms] OR "cambodia"[All Fields] OR "cambodia s"[All Fields]) OR ("cameroon"[MeSH Terms] OR "cameroon"[All Fields] OR "cameroons"[All Fields] OR "cameroon s"[All Fields]) OR ("central african republic"[MeSH Terms] OR ("central"[All Fields] AND "african"[All Fields] AND "republic"[All Fields]) OR "central african republic"[All Fields]) OR "C.A.R"[All Fields] OR ("chad"[MeSH Terms] OR "chad"[All Fields]) OR ("comoros"[MeSH Terms] OR "comoros"[All Fields] OR "comoro"[All Fields]) OR ("democratic republic of the congo"[MeSH Terms] OR ("democratic"[All Fields] AND "republic"[All Fields] AND "congo"[All Fields]) OR "democratic republic of the congo"[All Fields]) OR "DRC"[All Fields] OR (("democrat"[All Fields] OR "democratic"[All Fields] OR "democratically"[All Fields] OR "democratization"[All Fields] OR "democratize"[All Fields] OR "democratized"[All Fields] OR "democratizing"[All Fields] OR "democrats"[All Fields]) AND ("republic"[All Fields] OR "republic s"[All Fields] OR "republics"[All Fields]) AND ("congo"[MeSH Terms] OR "congo"[All Fields])) OR ("congo"[MeSH Terms] OR "congo"[All Fields] OR ("republic"[All Fields] AND "congo"[All Fields]) OR "republic of the congo"[All Fields]) OR ("costa rica"[MeSH Terms] OR ("costa"[All Fields] AND "rica"[All Fields]) OR "costa rica"[All Fields]) OR ("cote d ivoire"[MeSH Terms] OR ("cote"[All Fields] AND "d ivoire"[All Fields]) OR "cote d ivoire"[All Fields]) OR ("cote d ivoire"[MeSH Terms] OR ("cote"[All Fields] AND "d ivoire"[All Fields]) OR "cote d ivoire"[All Fields]) OR ("cote d ivoire"[MeSH Terms] OR ("cote"[All Fields] AND "d ivoire"[All Fields]) OR "cote d ivoire"[All Fields] OR ("ivory"[All Fields] AND "coast"[All Fields]) OR "ivory coast"[All Fields]) OR ("djibouti"[MeSH Terms] OR "djibouti"[All Fields]) OR ("egypt"[MeSH Terms] OR "egypt"[All Fields] OR ("arab"[All Fields] AND "republic"[All Fields] AND "egypt"[All Fields]) OR "arab republic of egypt"[All Fields]) OR ("el salvador"[MeSH Terms] OR ("el"[All Fields] AND "salvador"[All Fields]) OR "el salvador"[All Fields]) OR (("republic"[All Fields] OR "republic s"[All Fields] OR "republics"[All Fields]) AND ("el salvador"[MeSH Terms] OR ("el"[All Fields] AND "salvador"[All Fields]) OR "el salvador"[All Fields])) OR ("eritrea"[MeSH Terms] OR "eritrea"[All Fields]) OR ("eswatini"[MeSH Terms] OR "eswatini"[All Fields]) OR ("ethiopia"[MeSH Terms] OR "ethiopia"[All Fields] OR "ethiopia s"[All Fields]) OR ("gambia"[MeSH Terms] OR "gambia"[All Fields] OR "gambia s"[All Fields]) OR ("gambia"[MeSH Terms] OR "gambia"[All Fields] OR "the gambia"[All Fields]) OR ("ghana"[MeSH Terms] OR "ghana"[All Fields] OR "ghana s"[All Fields]) OR ("guinea"[MeSH Terms] OR "guinea"[All Fields] OR "guinea s"[All Fields] OR "guineas"[All Fields]) OR ("guinea bissau"[MeSH Terms] OR "guinea bissau"[All Fields] OR ("guinea"[All Fields] AND "bissau"[All Fields]) OR "guinea bissau"[All Fields]) OR ("haiti"[MeSH Terms] OR "haiti"[All Fields] OR "haiti s"[All Fields]) OR ("honduras"[MeSH Terms] OR "honduras"[All Fields]) OR ("india"[MeSH Terms] OR "india"[All Fields] OR "india s"[All Fields] OR "indias"[All Fields]) OR ("indonesia"[MeSH Terms] OR "indonesia"[All Fields] OR "indonesia s"[All Fields] OR "indonesias"[All Fields]) OR ("iran"[MeSH Terms] OR "iran"[All Fields] OR ("islamic"[All Fields] AND "republic"[All Fields] AND "iran"[All Fields]) OR "islamic republic of iran"[All Fields]) OR ("iran"[MeSH Terms] OR "iran"[All Fields]) OR ("persia"[MeSH Terms] OR "persia"[All Fields]) OR ("kenya"[MeSH Terms] OR "kenya"[All Fields] OR "kenya s"[All Fields]) OR ("micronesia"[MeSH Terms] OR "micronesia"[All Fields] OR "kiribati"[All Fields]) OR ("democratic people s republic of korea"[MeSH Terms] OR ("democratic"[All Fields] AND "people s"[All Fields] AND "republic"[All Fields] AND "korea"[All Fields]) OR "democratic people s republic of korea"[All Fields]) OR ("kyrgyzstan"[MeSH Terms] OR "kyrgyzstan"[All Fields] OR ("kyrgyz"[All Fields] AND "republic"[All Fields]) OR "kyrgyz republic"[All Fields]) OR ("kyrgyzstan"[MeSH Terms] OR "kyrgyzstan"[All Fields]) OR ("laos"[MeSH Terms] OR "laos"[All Fields]) OR ("laos"[MeSH Terms] OR "laos"[All Fields] OR ("LAO"[All Fields] AND "people s"[All Fields] AND "democratic"[All Fields] AND "republic"[All Fields]) OR "lao people s democratic republic"[All Fields]) OR ("LAO"[All Fields] AND "PDR"[All Fields]) OR ("lebanon"[MeSH Terms] OR "lebanon"[All Fields] OR "lebanon s"[All Fields]) OR ("lesotho"[MeSH Terms] OR "lesotho"[All Fields]) OR ("liberia"[MeSH Terms] OR "liberia"[All Fields] OR "liberia s"[All Fields]) OR ("madagascar"[MeSH Terms] OR "madagascar"[All Fields] OR "madagascar s"[All Fields]) OR ("malawi"[MeSH Terms] OR "malawi"[All Fields] OR "malawi s"[All Fields]) OR ("mali"[MeSH Terms] OR "mali"[All Fields]) OR ("mauritania"[MeSH Terms] OR "mauritania"[All Fields]) OR ("micronesia"[MeSH Terms] OR "micronesia"[All Fields] OR ("federated"[All Fields] AND "states"[All Fields] AND "micronesia"[All Fields]) OR "federated states of micronesia"[All Fields]) OR ("micronesia"[MeSH Terms] OR "micronesia"[All Fields]) OR ("mongolia"[MeSH Terms] OR "mongolia"[All Fields] OR "mongolia s"[All Fields]) OR ("morocco"[MeSH Terms] OR "morocco"[All Fields]) OR ("mozambique"[MeSH Terms] OR "mozambique"[All Fields] OR "mozambique s"[All Fields]) OR ("myanmar"[MeSH Terms] OR "myanmar"[All Fields] OR "myanmar s"[All Fields] OR "myanmars"[All Fields]) OR ("nepal"[MeSH Terms] OR "nepal"[All Fields] OR "nepal s"[All Fields]) OR ("nicaragua"[MeSH Terms] OR "nicaragua"[All Fields] OR "nicaragua s"[All Fields]) OR ("niger"[MeSH Terms] OR "niger"[All Fields]) OR ("nigeria"[MeSH Terms] OR "nigeria"[All Fields] OR "nigeria s"[All Fields]) OR ("pakistan"[MeSH Terms] OR "pakistan"[All Fields] OR "pakistan s"[All Fields]) OR ("papua new guinea"[MeSH Terms] OR ("papua"[All Fields] AND "new"[All Fields] AND "guinea"[All Fields]) OR "papua new guinea"[All Fields]) OR "PNG"[All Fields] OR ("philippine"[All Fields] OR "philippines"[MeSH Terms] OR "philippines"[All Fields]) OR ("rwanda"[MeSH Terms] OR "rwanda"[All Fields] OR "rwanda s"[All Fields]) OR ("samoa"[MeSH Terms] OR "samoa"[All Fields] OR "samoas"[All Fields]) OR ("sao tome and principe"[MeSH Terms] OR ("sao"[All Fields] AND "tome"[All Fields] AND "principe"[All Fields]) OR "sao tome and principe"[All Fields]) OR ("sao tome and principe"[MeSH Terms] OR ("sao"[All Fields] AND "tome"[All Fields] AND "principe"[All Fields]) OR "sao tome and principe"[All Fields]) OR ("senegal"[MeSH Terms] OR "senegal"[All Fields] OR "senegal s"[All Fields]) OR ("serbia"[MeSH Terms] OR "serbia"[All Fields]) OR ("sierra leone"[MeSH Terms] OR ("sierra"[All Fields] AND "leone"[All Fields]) OR "sierra leone"[All Fields]) OR ("melanesia"[MeSH Terms] OR "melanesia"[All Fields] OR ("solomon"[All Fields] AND "islands"[All Fields]) OR "solomon islands"[All Fields]) OR ("somalia"[MeSH Terms] OR "somalia"[All Fields]) OR ("south sudan"[MeSH Terms] OR ("south"[All Fields] AND "sudan"[All Fields]) OR "south sudan"[All Fields]) OR ("sri lanka"[MeSH Terms] OR ("sri"[All Fields] AND "lanka"[All Fields]) OR "sri lanka"[All Fields]) OR ("sudan"[MeSH Terms] OR "sudan"[All Fields] OR "sudans"[All Fields] OR "sudan s"[All Fields]) OR ("syria"[MeSH Terms] OR "syria"[All Fields] OR "syria s"[All Fields]) OR ("syria"[MeSH Terms] OR "syria"[All Fields] OR ("syrian"[All Fields] AND "arab"[All Fields] AND "republic"[All Fields]) OR "syrian arab republic"[All Fields]) OR ("tajikistan"[MeSH Terms] OR "tajikistan"[All Fields]) OR ("tanzania"[MeSH Terms] OR "tanzania"[All Fields] OR "tanzania s"[All Fields]) OR ("timor leste"[MeSH Terms] OR "timor leste"[All Fields] OR ("timor"[All Fields] AND "leste"[All Fields]) OR "timor leste"[All Fields]) OR ("togo"[MeSH Terms] OR "togo"[All Fields]) OR ("tunisia"[MeSH Terms] OR "tunisia"[All Fields]) OR ("uganda"[MeSH Terms] OR "uganda"[All Fields] OR "uganda s"[All Fields]) OR ("ukraine"[MeSH Terms] OR "ukraine"[All Fields] OR "ukraine s"[All Fields]) OR ("uzbekistan"[MeSH Terms] OR "uzbekistan"[All Fields]) OR ("vanuatu"[MeSH Terms] OR "vanuatu"[All Fields]) OR ("vietnam"[MeSH Terms] OR "vietnam"[All Fields] OR "vietnam s"[All Fields]) OR (("middle east"[MeSH Terms] OR ("middle"[All Fields] AND "east"[All Fields]) OR "middle east"[All Fields] OR ("west"[All Fields] AND "bank"[All Fields]) OR "west bank"[All Fields]) AND "gaza"[All Fields]) OR (("yemeni"[All Fields] OR "yemenis"[All Fields]) AND ("republic"[All Fields] OR "republic s"[All Fields] OR "republics"[All Fields])) OR ("yemen"[MeSH Terms] OR "yemen"[All Fields] OR ("republic"[All Fields] AND "yemen"[All Fields]) OR "republic of yemen"[All Fields]) OR ("yemen"[MeSH Terms] OR "yemen"[All Fields]) OR ("zambia"[MeSH Terms] OR "zambia"[All Fields] OR "zambia s"[All Fields]) OR ("zimbabwe"[MeSH Terms] OR "zimbabwe"[All Fields] OR "zimbabwe s"[All Fields])) AND ("mortality"[MeSH Terms] OR (("excess"[All Fields] OR "excesses"[All Fields] OR "excessive"[All Fields] OR "excessively"[All Fields]) AND "mortalit*"[All Fields]) OR ("All-cause"[All Fields] AND ("mortality"[MeSH Terms] OR "mortality"[All Fields] OR "mortalities"[All Fields] OR "mortality"[MeSH Subheading])) OR (("excess"[All Fields] OR "excesses"[All Fields] OR "excessive"[All Fields] OR "excessively"[All Fields]) AND "death*"[All Fields]) OR (("addition"[All Fields] OR "additional"[All Fields] OR "additions"[All Fields] OR "additive"[All Fields] OR "additively"[All Fields] OR "additives"[All Fields] OR "additivities"[All Fields] OR "additivity"[All Fields]) AND "death*"[All Fields]) OR (("increase"[All Fields] OR "increased"[All Fields] OR "increases"[All Fields] OR "increasing"[All Fields] OR "increasings"[All Fields]) AND "mortalit*"[All Fields]) OR ("higher"[All Fields] AND ("mortality"[MeSH Terms] OR "mortality"[All Fields] OR "mortalities"[All Fields] OR "mortality"[MeSH Subheading])) OR (("excess"[All Fields] OR "excesses"[All Fields] OR "excessive"[All Fields] OR "excessively"[All Fields]) AND "fatalit*"[All Fields])) AND ("covid 19"[MeSH Terms] OR ("covid 19"[All Fields] OR "covid 19"[MeSH Terms] OR "covid 19 vaccines"[All Fields] OR "covid 19 vaccines"[MeSH Terms] OR "covid 19 serotherapy"[All Fields] OR "covid 19 serotherapy"[Supplementary Concept] OR "covid 19 nucleic acid testing"[All Fields] OR "covid 19 nucleic acid testing"[MeSH Terms] OR "covid 19 serological testing"[All Fields] OR "covid 19 serological testing"[MeSH Terms] OR "covid 19 testing"[All Fields] OR "covid 19 testing"[MeSH Terms] OR "sars cov 2"[All Fields] OR "sars cov 2"[MeSH Terms] OR "severe acute respiratory syndrome coronavirus 2"[All Fields] OR "ncov"[All Fields] OR "2019 ncov"[All Fields] OR (("coronavirus"[MeSH Terms] OR "coronavirus"[All Fields] OR "cov"[All Fields]) AND 2019/11/01:3000/12/31[Date - Publication])) OR ("sars cov 2"[MeSH Terms] OR "sars cov 2"[All Fields] OR "sars cov 2"[All Fields]) OR ("sars cov 2"[MeSH Terms] OR "sars cov 2"[All Fields] OR "severe acute respiratory syndrome coronavirus 2"[All Fields]) OR ("covid 19"[MeSH Terms] OR "covid 19"[All Fields] OR "covid 19 pandemic"[All Fields]) OR ("pandemic s"[All Fields] OR "pandemically"[All Fields] OR "pandemicity"[All Fields] OR "pandemics"[MeSH Terms] OR "pandemics"[All Fields] OR "pandemic"[All Fields]) OR "covid*"[All Fields] OR "coronaviru*"[All Fields] OR ("SARS"[All Fields] AND "coronavirus*"[All Fields]) OR "sars cov*"[All Fields]) |

**#Scopus**

| **Key concept** |  |
| --- | --- |
| **COVID-19** | TITLE-ABS-KEY ( ( *covid-19*  AND *[mesh]* )  OR  ( *covid-19* )  OR  ( *sars-cov-2* )  OR  ( *severe*  AND *acute*  AND *respiratory*  AND *syndrome*  AND *coronavirus*  *2* )  OR  ( *covid-19*  AND *pandemic* )  OR  ( *pandemic* )  OR  ( *covid** )  OR  ( *coronaviru** )  OR  ( *sars*  AND *coronavirus** )  OR  ( *sars-cov** ) ) |
| **Excess Mortality** | TITLE-ABS-KEY ( ( *mortality*  AND *[mesh]* )  OR  ( *excess*  AND *mortalit** )  OR  ( *all-cause*  AND *mortality* )  OR  ( *excess*  AND *death** )  OR  ( *additional*  AND *death** )  OR  ( *increase*  AND *mortalit** )  OR  ( *higher*  AND *mortality* )  OR  ( *excess*  AND *fatalit** ) ) |
| **Low-and-lower -middle income countries** | ( *developing*  AND *countries*  AND *[mesh]* )  OR  ( *least*  AND *developed*  AND *countries* )  OR  ( *ldc* )  OR  ( *developing*  AND *countries* )  OR  ( *low-income*  AND *countries* )  OR  ( *lic* )  OR  ( *lower-*  AND *middle-income*  AND *countries* )  OR  ( *lmc* )  OR  ( *low-and-lower middle*  AND *income*  AND *countries* )  OR  ( *llmic* )  OR  ( *afghanistan* )  OR  ( *algeria* )  OR  ( *angola* )  OR  ( *bangladesh* )  OR  ( *benin* )  OR  ( *bhutan* )  OR  ( *bolivia* )  OR  ( *burkina*  AND *faso* )  OR  ( *burundi* )  OR  ( *cabo*  AND *verde* )  OR  ( *cambodia* )  OR  ( *cameroon* )  OR  ( *central*  AND *african*  AND *republic* )  OR  ( *c.a.r* )  OR  ( *chad* )  OR  ( *comoros* )  OR  ( *democratic*  AND *republic*  AND *of*  AND *the*  AND *congo* )  OR  ( *drc* )  OR  ( *democratic*  AND *republic*  AND *congo* )  OR  ( *republic*  AND *of*  AND *the*  AND *congo* )  OR  ( *costa*  AND *rica* )  OR  ( *cote*  AND *d'ivoire* )  OR  ( *côte*  AND *d'ivoire* )  OR  ( *ivory*  AND *coast* )  OR  ( *djibouti* )  OR  ( *arab*  AND *republic*  AND *of*  AND *egypt* )  OR  ( *el*  AND *salvador* )  OR  ( *republic*  AND *el*  AND *salvador* )  OR  ( *eritrea* )  OR  ( *eswatini* )  OR  ( *ethiopia* )  OR  ( *gambia* )  OR  ( *the*  AND *gambia* )  OR  ( *ghana* )  OR  ( *guinea* )  OR  ( *guinea-bissau* )  OR  ( *haiti* )  OR  ( *honduras* )  OR  ( *india* )  OR  ( *indonesia* )  OR  ( *islamic*  AND *republic*  AND *of*  AND *iran* )  OR  ( *iran* )  OR  ( *persia* )  OR  ( *kenya* )  OR  ( *kiribati* )  OR  ( *democratic*  AND *people's*  AND *republic*  AND *of*  AND *korea* )  OR  ( *kyrgyz*  AND *republic* )  OR  ( *kyrgyzstan* )  OR  ( *laos* )  OR  ( *lao*  AND *people's*  AND *democratic*  AND *republic* )  OR  ( *lao*  AND *pdr* )  OR  ( *lebanon* )  OR  ( *lesotho* )  OR  ( *liberia* )  OR  ( *madagascar* )  OR  ( *malawi* )  OR  ( *mali* )  OR  ( *mauritania* )  OR  ( *federated*  AND *states*  AND *of*  AND *micronesia* )  OR  ( *micronesia* )  OR  ( *mongolia* )  OR  ( *morocco* )  OR  ( *mozambique* )  OR  ( *myanmar* )  OR  ( *nepal* )  OR  ( *nicaragua* )  OR  ( *niger* )  OR  ( *nigeria* )  OR  ( *pakistan* )  OR  ( *papua*  AND *new*  AND *guinea* )  OR  ( *png* )  OR  ( *philippines* )  OR  ( *rwanda* )  OR  ( *samoa* )  OR  ( *são*  AND *tomé*  AND  *príncipe* )  OR  ( *sao*  AND *tome*  AND  *principe* )  OR  ( *senegal* )  OR  ( *serbia* )  OR  ( *sierra*  AND *leone* )  OR  ( *solomon*  AND *islands* )  OR  ( *somalia* )  OR  ( *south*  AND *sudan* )  OR  ( *sri*  AND *lanka* )  OR  ( *sudan* )  OR  ( *syria* )  OR  ( *syrian*  AND *arab*  AND *republic* )  OR  ( *tajikistan* )  OR  ( *tanzania* )  OR  ( *timor-leste* )  OR  ( *togo* )  OR  ( *tunisia* )  OR  ( *uganda* )  OR  ( *ukraine* )  OR  ( *uzbekistan* )  OR  ( *vanuatu* )  OR  ( *vietnam* )  OR  ( *west*  AND *bank*  AND  *gaza* )  OR  ( *yemeni*  AND *republic* )  OR  ( *republic*  AND *of*  AND *yemen* )  OR  ( *yemen* )  OR  ( *zambia* )  OR  ( *zimbabwe* ) |
| **ALL CONCEPTS COMBINED** | ( TITLE-ABS-KEY ( ( *covid-19*  AND *[mesh]* )  OR  ( *covid-19* )  OR  ( *sars-cov-2* )  OR  ( *severe*  AND *acute*  AND *respiratory*  AND *syndrome*  AND *coronavirus*  *2* )  OR  ( *covid-19*  AND *pandemic* )  OR  ( *pandemic* )  OR  ( *covid** )  OR  ( *coronaviru** )  OR  ( *sars*  AND *coronavirus** )  OR  ( *sars-cov** ) ) )  AND  ( TITLE-ABS-KEY ( ( *mortality*  AND *[mesh]* )  OR  ( *excess*  AND *mortalit** )  OR  ( *all-cause*  AND *mortality* )  OR  ( *excess*  AND *death** )  OR  ( *additional*  AND *death** )  OR  ( *increase*  AND *mortalit** )  OR  ( *higher*  AND *mortality* )  OR  ( *excess*  AND *fatalit** ) ) )  AND  ( ( *developing*  AND *countries*  AND *[mesh]* )  OR  ( *least*  AND *developed*  AND *countries* )  OR  ( *ldc* )  OR  ( *developing*  AND *countries* )  OR  ( *low-income*  AND *countries* )  OR  ( *lic* )  OR  ( *lower-*  AND *middle-income*  AND *countries* )  OR  ( *lmc* )  OR  ( *low-and-lower-middle*  AND *income*  AND *countries* )  OR  ( *llmic* )  OR  ( *afghanistan* )  OR  ( *algeria* )  OR  ( *angola* )  OR  ( *bangladesh* )  OR  ( *benin* )  OR  ( *bhutan* )  OR  ( *bolivia* )  OR  ( *burkina*  AND *faso* )  OR  ( *burundi* )  OR  ( *cabo*  AND *verde* )  OR  ( *cambodia* )  OR  ( *cameroon* )  OR  ( *central*  AND *african*  AND *republic* )  OR  ( *c.a.r* )  OR  ( *chad* )  OR  ( *comoros* )  OR  ( *democratic*  AND *republic*  AND *of*  AND *the*  AND *congo* )  OR  ( *drc* )  OR  ( *democratic*  AND *republic*  AND *congo* )  OR  ( *republic*  AND *of*  AND *the*  AND *congo* )  OR  ( *costa*  AND *rica* )  OR  ( *cote*  AND *d'ivoire* )  OR  ( *côte*  AND *d'ivoire* )  OR  ( *ivory*  AND *coast* )  OR  ( *djibouti* )  OR  ( *arab*  AND *republic*  AND *of*  AND *egypt* )  OR  ( *el*  AND *salvador* )  OR  ( *republic*  AND *el*  AND *salvador* )  OR  ( *eritrea* )  OR  ( *eswatini* )  OR  ( *ethiopia* )  OR  ( *gambia* )  OR  ( *the*  AND *gambia* )  OR  ( *ghana* )  OR  ( *guinea* )  OR  ( *guinea-bissau* )  OR  ( *haiti* )  OR  ( *honduras* )  OR  ( *india* )  OR  ( *indonesia* )  OR  ( *islamic*  AND *republic*  AND *of*  AND *iran* )  OR  ( *iran* )  OR  ( *persia* )  OR  ( *kenya* )  OR  ( *kiribati* )  OR  ( *democratic*  AND *people's*  AND *republic*  AND *of*  AND *korea* )  OR  ( *kyrgyz*  AND *republic* )  OR  ( *kyrgyzstan* )  OR  ( *laos* )  OR  ( *lao*  AND *people's*  AND *democratic*  AND *republic* )  OR  ( *lao*  AND *pdr* )  OR  ( *lebanon* )  OR  ( *lesotho* )  OR  ( *liberia* )  OR  ( *madagascar* )  OR  ( *malawi* )  OR  ( *mali* )  OR  ( *mauritania* )  OR  ( *federated*  AND *states*  AND *of*  AND *micronesia* )  OR  ( *micronesia* )  OR  ( *mongolia* )  OR  ( *morocco* )  OR  ( *mozambique* )  OR  ( *myanmar* )  OR  ( *nepal* )  OR  ( *nicaragua* )  OR  ( *niger* )  OR  ( *nigeria* )  OR  ( *pakistan* )  OR  ( *papua*  AND *new*  AND *guinea* )  OR  ( *png* )  OR  ( *philippines* )  OR  ( *rwanda* )  OR  ( *samoa* )  OR  ( *são*  AND *tomé*  AND  *príncipe* )  OR  ( *sao*  AND *tome*  AND  *principe* )  OR  ( *senegal* )  OR  ( *serbia* )  OR  ( *sierra*  AND *leone* )  OR  ( *solomon*  AND *islands* )  OR  ( *somalia* )  OR  ( *south*  AND *sudan* )  OR  ( *sri*  AND *lanka* )  OR  ( *sudan* )  OR  ( *syria* )  OR  ( *syrian*  AND *arab*  AND *republic* )  OR  ( *tajikistan* )  OR  ( *tanzania* )  OR  ( *timor-leste* )  OR  ( *togo* )  OR  ( *tunisia* )  OR  ( *uganda* )  OR  ( *ukraine* )  OR  ( *uzbekistan* )  OR  ( *vanuatu* )  OR  ( *vietnam* )  OR  ( *west*  AND *bank*  AND  *gaza* )  OR  ( *yemeni*  AND *republic* )  OR  ( *republic*  AND *of*  AND *yemen* )  OR  ( *yemen* )  OR  ( *zambia* )  OR  ( *zimbabwe* ) ) |

**#Cochrane Library**

| **Key concepts** | **Search details** |
| --- | --- |
| **COVID-19** | (COVID-19) OR (SARS-CoV-2) OR (Severe Acute Respiratory Syndrome Coronavirus 2) OR (COVID-19 pandemic) OR (Pandemic) OR (COVID*) OR (Coronaviru*) OR (SARS Coronavirus*) OR (SARS-COV*) in Title Abstract Keyword - (Word variations have been searched) |
| **Excess mortality** | (Excess mortalit*) OR (All-cause mortality) OR (excess death*) OR (Additional death*) OR (Increase mortalit*) OR (higher mortality) OR (Excess fatalit*) in Title Abstract Keyword - (Word variations have been searched) |
| **Low and lower middle income countries** | (Least developed countries) OR (LDC) OR (Developing Countries) OR (Low income countries) OR (LIC) OR (Lower Middle-income countries) OR (LMC) OR (Low and lower middle income countries) OR (LLMIC) OR (Afghanistan) OR (Algeria) OR (Angola) OR (Bangladesh) OR (Benin) OR (Bhutan) OR (Bolivia) OR (Burkina Faso) OR (Burundi) OR (Cabo Verde) OR (Cambodia) OR (Cameroon) OR (Central African Republic) OR (C.A.R) OR (Chad) OR (Comoros) OR (Democratic Republic of the Congo) OR (DRC) OR (Democratic Republic Congo) OR (Republic of the Congo) OR (Costa Rica) OR (Cote d'Ivoire) OR (Côte d'Ivoire) OR (Ivory Coast) OR (Djibouti) OR (Arab Republic of Egypt) OR (El Salvador) OR (Republic El Salvador) OR (Eritrea) OR (Eswatini) OR (Ethiopia) OR (Gambia) OR (The Gambia) OR (Ghana) OR (Guinea) OR (Guinea-Bissau) OR (Haiti) OR (Honduras) OR (India) OR (Indonesia) OR (Islamic Republic of Iran) OR (Iran) OR (Persia) OR (Kenya) OR (Kiribati) OR (democratic People's Republic of Korea) OR (Kyrgyz republic) OR (Kyrgyzstan) OR (Laos) OR (Lao People's Democratic Republic) or (LAO PDR) OR (Lebanon) OR (Lesotho) OR (Liberia) OR(Madagascar) OR (Malawi) OR (Mali) OR (Mauritania) OR (Federated States of Micronesia) OR (Micronesia) OR (Mongolia) OR (Morocco) OR (Mozambique) OR (Myanmar) OR (Nepal) OR (Nicaragua) OR (Niger) OR (Nigeria) OR (Pakistan) OR (Papua New Guinea) OR (PNG) OR (Philippines) OR (Rwanda) OR (Samoa) OR (São Tomé and Príncipe) OR (Sao Tome and Principe) OR (Senegal) OR (Serbia) OR (Sierra Leone) OR (Solomon islands) OR (Somalia) OR (South sudan) OR (Sri lanka) OR (Sudan) OR (Syria) OR (Syrian Arab republic) OR (Tajikistan) OR (Tanzania) OR (Timor-leste) OR (Togo) OR (Tunisia) OR (Uganda) OR (Ukraine) OR (Uzbekistan) OR (Vanuatu) OR (Vietnam) OR (West bank and gaza) OR (Yemeni Republic) OR (Republic of Yemen) OR (Yemen) OR (Zambia) OR (Zimbabwe) in Title Abstract Keyword - (Word variations have been searched) |

**#Google Scholar**

**allintitle:** "Excess mortality" OR "All cause mortality" OR "excess deaths" OR "Additional deaths"

AND (Least developed countries) OR (LDC) OR (Low-income countries) OR (LIC) OR (Lower- Middle-income countries) OR (LMC) OR (Low-and-lower-middle income countries) OR (LLMIC) OR (Afghanistan) OR (Algeria) OR (Angola) OR (Bangladesh) OR (Benin) OR (Bhutan) OR (Bolivia) OR (Burkina Faso) OR (Burundi) OR (Cabo Verde) OR (Cambodia) OR (Cameroon) OR (Central African Republic) OR (C.A.R) OR (Chad) OR (Comoros) OR (Democratic Republic of the Congo) OR (DRC) OR (Costa Rica) OR (Cote d'Ivoire) OR (Côte d'Ivoire) OR (Ivory Coast) OR (Djibouti) OR (Arab Republic of Egypt) OR (El Salvador) OR (Republic El Salvador) OR (Eritrea) OR (Eswatini) OR (Ethiopia) OR (Gambia) OR (Ghana) OR (Guinea) OR (Guinea-Bissau) OR (Haiti) OR (Honduras) OR (India) OR (Indonesia) OR (Islamic Republic of Iran) OR Iran OR Persia OR Kenya OR Kiribati OR democratic People's Republic of Korea OR Kyrgyz republic OR Kyrgyzstan OR Laos OR Lao People's Democratic Republic OR LAO PDR OR Lebanon OR Lesotho OR (Liberia) OR (Madagascar) OR (Malawi) OR (Mali) OR (Mauritania) OR (Federated States of Micronesia) OR (Micronesia) OR (Mongolia) OR (Morocco) OR (Mozambique) OR (Myanmar) OR (Nepal) OR (Nicaragua) OR (Niger) OR (Nigeria) OR (Pakistan) OR (Papua New Guinea) OR (PNG) OR (Philippines) OR (Rwanda) OR (Samoa) OR (São Tomé and Príncipe) OR (Sao Tome and Principe) OR (Senegal) OR (Serbia) OR (Sierra Leone) OR (Solomon islands) OR (Somalia) OR (South sudan) OR (Sri lanka) OR (Sudan) OR (Syria) OR (Syrian Arab republic) OR (Tajikistan) OR (Tanzania) OR (Timor-leste) OR (Togo) OR (Tunisia) OR (Uganda) OR (Ukraine) OR (Uzbekistan) OR (Vanuatu) OR (Vietnam) OR (West bank and gaza) OR (Yemeni Republic) OR (Republic of Yemen) OR (Yemen) OR (Zambia) OR (Zimbabwe)
